# Risk factors for breast cancer development by tumor characteristics among women with benign breast disease

**DOI:** 10.1186/s13058-021-01410-1

**Published:** 2021-03-18

**Authors:** Jonine D. Figueroa, Gretchen L. Gierach, Máire A. Duggan, Shaoqi Fan, Ruth M. Pfeiffer, Yihong Wang, Roni T. Falk, Olivier Loudig, Mustapha Abubakar, Mindy Ginsberg, Teresa M. Kimes, Kathryn Richert-Boe, Andrew G. Glass, Thomas E. Rohan

**Affiliations:** 1 National Cancer Institute, National Institutes of Health, Division of Cancer Epidemiology and Genetics, Bethesda, MD USA; 2 The Usher Institute, Old Medical School, The University of Edinburgh, Teviot Place, Edinburgh, UK; 3 CRUK Edinburgh Centre, The University of Edinburgh, Edinburgh, UK; 4 Department of Pathology and Laboratory Medicine, Cumming School of Medicine, University of Calgary, Alberta Calgary, Canada; 5 Warren Alpert Medical School of Brown University, Providence, RI USA; 6 Center for Discovery and Innovation (CDI), Hackensack Meridian Health, Nutley, NJ USA; 7 Albert Einstein College of Medicine, Jack and Pearl Resnick Campus, 1300 Morris Park Avenue, Belfer Building, Room 1301, Bronx, NY 10461 USA; 8 Kaiser Permanente Center for Health Research, Portland, OR USA

**Keywords:** Benign breast disease, Estrogen receptor, Columnar cell lesions, Breast cancer, Tumor characteristics, Epidemiology

## Abstract

**Background:**

Among women diagnosed with invasive breast cancer, 30% have a prior diagnosis of benign breast disease (BBD). Thus, it is important to identify factors among BBD patients that elevate invasive cancer risk. In the general population, risk factors differ in their associations by clinical pathologic features; however, whether women with BBD show etiologic heterogeneity in the types of breast cancers they develop remains unknown.

**Methods:**

Using a nested case-control study of BBD and breast cancer risk conducted in a community healthcare plan (Kaiser Permanente Northwest), we assessed relationships of histologic features in BBD biopsies and patient characteristics with subsequent breast cancer risk and tested for heterogeneity of associations by estrogen receptor (ER) status, tumor grade, and size. The study included 514 invasive breast cancer cases (median follow-up of 9 years post-BBD diagnosis) and 514 matched controls, diagnosed with proliferative or non-proliferative BBD between 1971 and 2006, with follow-up through mid-2015. Odds ratios (ORs) and 95% confidence intervals (CIs) were obtained using multivariable polytomous logistic regression models.

**Results:**

Breast cancers were predominantly ER-positive (86%), well or moderately differentiated (73%), small (74% < 20 mm), and stage I/II (91%). Compared to patients with non-proliferative BBD, proliferative BBD with atypia conferred increased risk for ER-positive cancer (OR = 5.48, 95% CI = 2.14–14.01) with only one ER-negative case, *P*-heterogeneity = 0.45. The presence of columnar cell lesions (CCLs) at BBD diagnosis was associated with a 1.5-fold increase in the risk of both ER-positive and ER-negative tumors, with a 2-fold increase (95% CI = 1.21–3.58) observed among postmenopausal women (56%), independent of proliferative BBD status with and without atypia. We did not identify statistically significant differences in risk factor associations by tumor grade or size.

**Conclusion:**

Most tumors that developed after a BBD diagnosis in this cohort were highly treatable low-stage ER-positive tumors. CCL in BBD biopsies may be associated with moderately increased risk, independent of BBD histology, and irrespective of ER status.

**Supplementary Information:**

The online version contains supplementary material available at 10.1186/s13058-021-01410-1.

## Introduction

It is well established that benign breast diseases (BBDs) increase the risk of breast cancer in women [1]. In the USA, biopsy-confirmed BBD diagnoses are 4 times more common than invasive breast cancers, affecting ~ 1 million women annually. BBDs include a range of pathologies with approximately 30% comprising proliferative BBD lesions (PDWA, proliferative disease, i.e., without atypia) and 65% encompassing non-proliferative benign lesions [2, 3]. Proliferative BBD with atypia (atypical hyperplasia) represents 3–4% of BBD diagnoses, has been associated with a 4–5-fold increased breast cancer (BC) risk [3, 4], and is considered as an indication for possible chemoprevention [5].

Since women diagnosed with BBD comprise a large proportion of future breast cancer cases (~ 30%), it is important to identify factors associated with subsequent BC [3]. Having undergone a clinically indicated breast biopsy, women with BBD provide the opportunity for evaluation of whether histopathologic features increase BC risk independently of other patient characteristics. Providing accurate invasive BC risk estimates for BBD patients could improve clinical management of this large group of women, including determination of whether watchful waiting, surgery, or chemoprevention should be offered [3]. In a model that predicted overall BC risk for BBD patients, key risk factors were parity/age at first birth and family history of breast cancer, as well as histologic features, including columnar cell lesions (CCLs), radial scars, sclerosing adenosis, and lobular involution [6]. Within the Kaiser BBD study, previous analyses examined associations of lifestyle, reproductive, and pathologic characteristics that are associated with subsequent risk of breast cancer [7, 8]; however, whether risks associated with these factors are similar or vary by the characteristics of the tumors diagnosed among BBD patients is not well understood and has not been previously assessed.

Breast cancer is a heterogeneous disease, with data strongly supporting different associations of risk factors such as parity and genetic susceptibility loci by tumor subtypes defined by hormone receptor status and other clinical/pathologic features [9–12]. Whether similar etiologic heterogeneity exists for breast cancer arising in BBD patients is unknown, and to date, very few cohort studies have been able to assess this hypothesis [7, 13, 14]. We therefore assessed patient and BBD histopathological features associated with BC risk and evaluated whether relationships varied by tumor characteristics.

## Methods

### Study population

We utilized data from a previously designed case-control study of BBD and breast cancer risk [7], nested within a BBD cohort from Kaiser Permanente Northwest (KPNW), which provides medical care for approximately ½ million members located in Southwest Washington and Northwest Oregon. The source population was comprised of women who had biopsy-confirmed BBD diagnosed between August 3, 1971, and December 31, 2006 (*N* = 15,395; age range 21–85 years); one control had a BBD diagnosis in 2012. Invasive breast cancer diagnoses were obtained through record linkage with the KPNW Tumor Registry, as previously described [7]. Briefly, cases were defined as women diagnosed with invasive breast cancer at least 1 year after BBD diagnosis and with no prior history of in situ lesions. The follow-up rate for the KPNW Tumor Registry since its inception in 1960 has been excellent, accounting for 98% of patients (living or dead) even if they are no longer health plan members. Controls were women with a biopsy for BBD who were alive but had not developed breast cancer during the same follow-up calendar period as that for the corresponding cases. For each case, a control case was randomly selected using risk-set sampling with replacement, matched at age at diagnosis of BBD (± 1 year) and, implicitly, given the risk-set sampling, on duration of membership of KPNW [15]. In addition, each control was required not to have undergone a mastectomy before the date of diagnosis of breast cancer for her matched case. If a selected control did not have breast tissue for evaluation or had no risk factor information, a replacement control was selected. There were 514 cases and 514 matched controls available for these analyses.

### Tumor characteristics

We obtained information on the following variables from the KPNW Tumor Registry: date of invasive breast cancer diagnosis, date of death, tumor histology and behavior using ICD-O coding, grade, AJCC clinical staging variable, tumor size, and lymph node status. Immunohistochemistry (IHC) data on key markers were obtained for ER (which has been measured on cases since the mid-1970s), PR (first measured in 1983), and HER2 status (first measured in 1988). The percentage of tumors analyzed for ER status increased from 0% in 1980 to 18% in 1989, 54% in 1994, and 85% in 2006; HER2 status was available on 43% of tumors until 2001 and on 68% from 2002 onward.

### Risk factor information

Risk factor data were captured using routine medical records, which include information on clinic visits, prescriptions, operations, and laboratory testing. These data were linked using the KPNW unique health record numbers to identify each individual member. From these records, data were available for various exposures. We focused on established or suspected breast cancer risk factors, specifically family history of breast cancer, age at menarche, parity, age at first birth, body mass index (BMI) at benign biopsy, menopausal status, and menopausal hormone use. KPNW has a well-characterized mammography screening program, and by 1993, more than 75% of women over 45 had had a screening mammogram [16]. For this reason, we also report characteristics stratified by this date (Supplemental Table 1).

BBD histology was assessed according to the Page classification criteria [1], as follows: proliferative disease with atypia (ADH), if atypical hyperplasia (either ductal or lobular) was present; proliferative disease without atypia, if epithelial hyperplasia without atypia (either moderate or florid) OR fibroadenoma (either complex-no atypia or complex-atypia) OR sclerosing adenosis OR radial scar OR papilloma was present; non-proliferative, if non-proliferative lesion (either cysts, fibrosis, or apocrine metaplasia) OR mild epithelial hyperplasia without atypia OR fibroadenoma was present. Pathologist assessment of biopsies was blinded to case-control status as previously described [8]. CCL pathology and terminal duct lobular unit (TDLU) involution status were additionally ascertained on digitized BBD H&E images using the Aperio Scanscope system. Furthermore, as involution status is not typically reported in clinical pathology, we obtained more detailed semi-quantitative measures of TDLU involution previously reported to be associated with breast cancer risk among BBD patients (see Supplemental Methods).

### Multiple imputation

We used multiple imputation to impute missing data for risk factors. The largest amount of missingness (21%) was seen for the age at menarche variable (see Supplemental Methods and Appendix A). We did not impute missing data on MHT use, as the percentage of missingness was too large (45%) to allow for stable imputation. All variables that were correlated or associated with the outcome variables or that were potentially related to the missingness of other imputed variables were included in the imputation models (see Supplemental Methods) [17, 18]. Variables were imputed as continuous and later categorized for further analyses. Multiple imputations were performed using IVEware (0.3 version, https://www.src.isr.umich.edu/software/iveware-documentation/iveware-with-sas/). Details of imputation steps are delineated in a flow chart (Supplementary Figure 1 and Appendix A in Supplemental Material). All calculations presented in this paper were conducted for 5 imputed datasets separately; estimates from the different imputed datasets were combined and variances were computed using Rubin’s formula as implemented in SAS 9.4, PROC MIANALYZE.

### Statistical analysis

Descriptive statistics of demographic and tumor characteristics by calendar year of BBD diagnosis and age at breast cancer diagnosis were assessed using chi-squared or Fisher’s exact tests. Conditional logistic regression and unconditional logistic models adjusted for the matching factors yielded similar estimates (Supplemental Table 2). We therefore present odds ratios (ORs) and 95% confidence intervals (CIs) for demographic, reproductive, or tissue factors (explanatory variables) for overall breast cancer risk from unconditional regression models. The unconditional logistic regression models included matching factors, as well as continuous age at BBD diagnosis and follow-up period from BBD diagnosis to breast cancer diagnosis; other key risk factors included in the models were family history of breast cancer in 1st-degree relatives, history of bilateral oophorectomy, and parity. For the main analysis to determine associations by breast cancer subtype (comparing ER, PR, and HER2 status or clinicopathologically defined subtypes), we used polytomous logistic regression models adjusted for matching factors and using the same variables as for the overall BC model. Heterogeneity between factors was assessed using polytomous logistic regression analyses restricted to cases (case-only analyses) with the tumor characteristics (ER, tumor size, and grade) as the outcome variable. Models were also stratified by menopausal status. A *P* ≤ 0.05 was considered statistically significant and all tests were two-sided. All analyses were performed using SAS V9.4.

## Results

### Tumor characteristics by patient characteristics, age at breast cancer diagnosis, and BBD calendar year at diagnosis

Characteristics of the BBD patients are detailed in Supplemental Tables 1 and 2. The median age of BBD diagnosis was 51.5 years and over 60% of cases were diagnosed between 1980 and 1999. As mammography screening became more common after 1993, we observed an increased frequency of proliferative disease without atypia (38.5 vs 27.6%) and with atypia (7.3 vs 4.0%, Supplemental Table 1) subsequent to 1993. We also observed increased detection of prevalent breast cancer and a stage shift, with a 12% increase in diagnosed stage I tumors after 1993 (Supplemental Table 1). The median follow-up between BBD and breast cancer diagnosis was 9.0 years (IQR = 4.4, 15.8 years), with a median age at breast cancer diagnosis of 62.7 years. Descriptive characteristics of the BBD patients subsequently diagnosed with breast cancer, overall, and stratified by age at breast cancer diagnosis, are presented in Table 1. Most cases were older than 50 years (87%) at diagnosis. Breast cancers were mostly diagnosed from 1996 to 2005, and 55% of cases were diagnosed within 10 years of their initial BBD diagnosis.

**Table 1:** Characteristics of BBD histology among breast cancer cases, overall and by age and ER status at breast cancer diagnosis (*N* = 514)

			Age at breast cancer diagnosis					ER status				
	Cases		≤ 50 years (***N*** = 69)		> 50 years (***N*** = 445)		***P***^**a**^	ER+ (***N*** = 391)		ER− (***N*** = 64)		***P***^**a**^
	***N***	%	***N***	%	***N***	%		***N***	%	***N***	%	
**Age at BBD, years**												
< 40	90	17.5	41	59.4	49	11.0	**< 0.0001**	61	15.6	13	20.3	0.83
40–49	141	27.4	28	40.6	113	25.4		113	28.9	17	26.6	
50–59	145	28.2	0	0	145	32.6		111	28.4	19	29.7	
60–69	86	16.7	0	0	86	9.3		65	16.6	8	12.5	
≥ 70	52	10.1	0	0	52	11.7		41	10.5	7	10.9	
Mean (SD)	52.2 (12.5)		37.5 (6.9)		54.4 (11.6)			52.4 (12.5)		51.7 (12.9)		
Median (IQR)	51.5 (42.6, 60.8)		39.0 (34.9, 42.9)		53.6 (46.1, 62.1)			51.7 (42.8, 61.0)		50.6 (42.8, 59.8)		
**Year of BBD diagnosis**							0.062					0.92
1971–1979	95	18.5	19	27.5	76	17.1		50	12.8	10	15.6	
1980–1989	173	33.7	16	23.2	157	35.3		134	34.3	20	31.3	
1990–1999	161	31.3	25	36.2	136	30.6		134	34.3	22	34.4	
2000–2006^#^	85	16.5	9	13.0	76	17.1		73	18.7	12	18.8	
**BBD histology**							0.22					0.39
Normal/non-proliferative	324	63.0	50	72.5	274	61.6		243	62.2	43	67.2	
Proliferative without atypia	163	31.7	16	23.2	147	33.3		127	32.5	20	31.3	
Proliferative with atypia	27	5.3	3	4.4	24	5.4		21	5.4	1	1.6	
**Year of breast cancer diagnosis**							**< 0.0001**					0.53
1973–1990	72	14.1	22	31.9	51	11.4		21	5.4	3	4.7	
1991–1995	68	13.2	10	14.5	58	13.0		49	12.5	12	18.8	
1996–2000	101	19.7	17	24.6	84	18.8		81	20.7	17	26.6	
2001–2005	116	22.6	11	15.9	106	23.7		104	26.6	12	18.8	
2006–2010	106	20.6	5	7.3	101	22.6		91	23.3	14	21.9	
2011–2013	51	9.9	4	5.8	47	10.6		45	11.5	6	9.4	
**Years from BBD to breast cancer diagnosis**							**< 0.0001**					0.55
≤ 10	284	55.3	54	78.3	230	51.7		198	50.6	35	54.7	
> 10	230	44.8	15	21.7	215	48.3		193	49.4	29	45.3	
Mean (SD)	10.8 (7.9)		7.3 (6)		11.4 (8)			11.6 (8)		10.5 (8.3)		
Median (IQR)	9.0 (4.4, 15.8)		5.6 (2.9, 9.4)		9.8 (4.7, 16.3)			10.0 (5.3, 16.4)		8.7 (3.3, 15.7)		
**Tumor size/mm**							0.13					0.63
< 10	143	28.9	26	37.7	117	27.5		90	23.9	16	26.2	
10–20	211	42.6	29	42.0	182	42.7		173	45.9	24	39.3	
> 20	141	28.5	14	20.3	127	29.8		114	30.2	21	34.4	
Missing	19		0		19			14		3		
**Tumor grade**^**c**^							0.14					**< 0.0001**
Well differentiated	141	34.7	14	31.8	127	34.9		134	39.3	5	8.6	
Moderately differentiated	159	38.9	13	29.6	146	40.1		149	43.7	6	10.3	
Poorly differentiated	108	26.4	17	38.6	91	25.0		58	17.0	47	81.0	
Not determined	106		25		81			50		6		
**ER**							**0.026**					–
Negative	64	14.1	12	24.5	52	12.8		–	–	–	–	
Positive	391	85.9	37	75.5	354	87.2		–	–	–	–	
Missing/unknown	59		20		39			–	–	–	–	
**PR**							0.53					**< 0.0001**
Negative	131	28.9	16	32.7	115	28.4		73	18.7	58	90.6	
Positive	323	71.2	33	67.4	290	71.6		317	81.3	6	9.4	
Missing/unknown	60		20		40			1		0		
**HER2**^**d**^							0.99^b^					**0.0028**
Negative	224	79.4	17	73.9	207	79.9		203	81.5	20	62.5	
Positive	50	17.7	4	17.4	46	17.8		38	15.3	12	37.5	
Equivocal	8	2.8	2	8.7	6	2.3		8	3.2	0	0.0	
Missing/unknown	232		46		186			142		32		
**Regional lymph nodes**							0.98					0.11
Negative	337	73.6	47	73.4	230	73.6		264	74.6	36	64.3	
Positive	121	26.4	17	26.6	104	26.4		90	25.4	20	35.7	
Missing	56		5		51			37		8		
**Tumor histology**							0.54^b^					0.14^b^
Ductal	439	83.5	59	85.5	370	83.2		322	82.4	54	84.4	
Lobular	48	9.3	8	11.6	40	9.0		39	10.0	4	6.3	
Mixed ductal/lobular	26	5.1	2	2.9	24	5.4		23	5.9	2	3.1	
Others	11	2.1	0	0.0	11	2.5		7	1.8	4	6.3	
**Tumor stage**							0.46^b^					0.30^b^
I	262	58.0	32	62.8	230	57.4		223	59.3	29	47.5	
II	150	33.2	13	25.5	137	34.2		120	31.9	25	41.0	
III	29	6.4	4	7.8	25	6.2		24	6.4	5	8.2	
IV	11	2.4	2	3.9	9	2.2		9	2.4	2	3.3	
Missing	62		18		44			15		3		

Among all cases, 13.4% were diagnosed with breast cancer by 50 years old versus 86.6% were diagnosed after age at 50 years. Fifty-nine cases who had missing ER status were excluded from analyses with ER status. *BBD* benign breast disease, *ER* estrogen receptor status, *HER2* human epidermal growth factor, *IQR* inter-quartile range, *PR* progesterone receptor status, *SD* standard deviation. ^a^*P*-values from the chi-square test except where noted; cases with missing tumor characteristics were excluded from reported percentage and tests; *P*-values less than 0.05 are in bold font. ^b^*P*-values from the Fisher exact test. ^c^Patients with tumor grade as “not determined” were excluded from analysis. ^d^Patients with equivocal HER2 were excluded from the analysis. Involution and columnar cell lesion associations were adjusted for all factors above the line indicated in OR*. ^#^One control had a BBD diagnosis in 2012

Tumors diagnosed in this population were predominantly small (71.6% ≤ 20 mm), well or moderately differentiated (73.6%), ER-positive (85.9%), PR-positive (71.2%), HER2-negative (79.4%), lymph node-negative (73.6%), and of ductal histology (83.5%). The invasive cases were overwhelmingly of low stage, with < 10% of cases being diagnosed as stage III or IV. As expected, ER status differed significantly by age at breast cancer diagnosis, with a higher proportion (24.5%) of ER-negative breast cancers diagnosed among women ≤ 50 compared to those older than 50 (12.8%). We also assessed whether there were differences in tumor characteristics by calendar period before and during/after 1993 [16]. Of the tumor characteristics evaluated, HER2 status showed a statistically significant difference by BBD diagnosis before and during/after 1993, with a higher proportion of HER2-negative cases diagnosed after 1993 compared to prior years (84.2 vs 73.9%, Supplemental Table 1). After 1993, significantly more of the tumors occurred among women ≥ 50 years of age, were of smaller size (10–20 mm; 44.4% during/after 1993 vs 34.8% before 1993), and had no or mild involution (48.93% during/after 1993 vs 36.56% before 1993, Supplemental Table 1). Histologic grade data were not routinely reported prior to 1993.

### Breast cancer risk factors among women with BBD

**Fig. 1:**
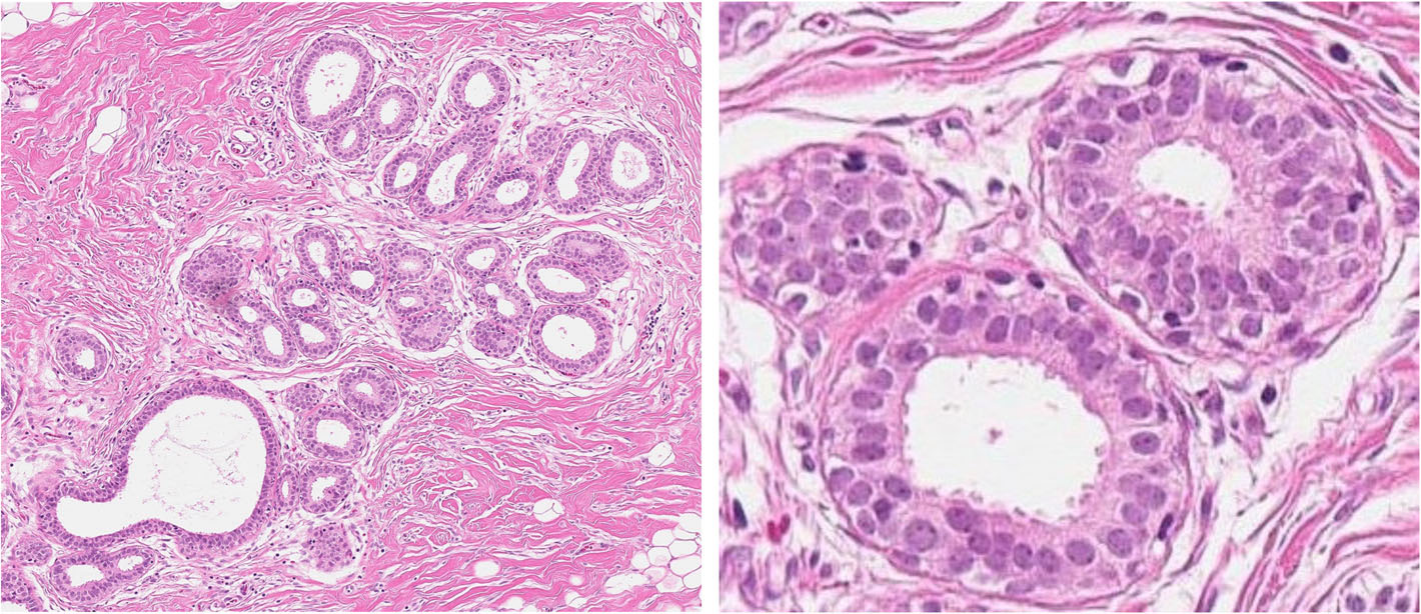
A representative hematoxylin and eosin stained breast biopsy × 200 μm image of columnar cell change showing dilated acini lined by a columnar epithelium demonstrating apical cytoplasmic snouts

Association results for all cases combined for established risk factors, including BBD characteristics, are shown in Supplemental Table 2. We found that younger age at first full-term birth and history of bilateral oophorectomy were inversely associated with breast cancer risk, whereas positive family history of breast cancer in a 1st-degree relative, increasing severity of BBD histology, and presence of CCL at BBD diagnosis were associated with increased breast cancer risk (Supplemental Table 2). A representative image of CCL is shown in Fig. 1. CCL with atypia, also known as flat epithelial atypia, is a more severe lesion that has been suggested to be associated with increased risk of breast cancer [3]; however, we were unable to assess this association in the present study as only 2 controls and 1 case had flat epithelial atypia. Lobular involution, which has been proposed in other BBD patient populations as a key risk factor for subsequent breast cancer [6, 19], was weakly inversely associated with breast cancer risk in our population [complete vs no involution, OR (95% CI) = 0.89 (0.65, 1.24)]. Neither radial scar nor sclerosing adenosis conferred a significant risk among those with proliferative BBD disease (data not shown).

### Breast cancer risk by ER status among women with BBD

Risk associations by ER status for patient characteristics and histologic features of BBD are presented in Table 2. Most tumors were ER-positive, which limited the power to detect heterogeneity; as a result, patterns of association observed for overall invasive breast cancer were generally consistent with those observed for ER-positive breast cancer risk. Having an age at first birth < 30 years was associated with reduced risk of ER-positive (OR = 0.69, 95% CI = 0.49–0.98), but not ER-negative (OR = 1.08, 95% CI = 0.51–2.30) breast cancer (*P*-heterogeneity = 0.24). Compared with patients with non-proliferative BBD, those with proliferative BBD with atypia had a greater than fivefold increased risk for ER-positive disease (OR = 5.48, 95% CI = 2.14–14.01). There was only one ER-negative case; hence, too few cases to provide a reliable estimate in this subgroup. After accounting for BBD histology, the presence of CCLs at BBD diagnosis was associated with a 1.5-fold increased risk for both ER-positive (95% CI = 1.03–2.29) and ER-negative (95% CI = 0.73–3.07) tumors (*P*-heterogeneity = 0.94).

**Table 2:** Multivariable associations between select patient characteristics and histologic features with breast cancer risk by ER status (*N* = 969)

Variable	Controls (***N*** = 514)		Cases, ER+ (***N*** = 391)		ER+ vs. controls	Cases, ER− (***N*** = 64)		ER− vs. controls	***P-***het^**†**^
	***N***^**a**^	%^**a**^	***N***^**a**^	%^**a**^	OR (95% CI)*	***N***^**a**^	%^**a**^	OR (95% CI)*	
**Age at first full-term birth/years**									
Nulliparous/≥ 30	108	20.9	88	27.6	1.00 (ref)	13	20.3	1.00 (ref)	0.24
< 30	406	79.1	231	72.4	**0.69 (0.49, 0.98)**	51	79.7	1.08 (0.51, 2.30)	
*P*-value					**0.038**			0.84	
**Family history of breast cancer**									
No	434	84.4	257	80.7	1.00 (ref)	50	77.5	1.00 (ref)	0.56
Yes	80	15.6	62	19.3	1.30 (0.89, 1.88)	14	22.5	1.60 (0.77, 3.33)	
*P*-value					0.17			0.20	
**History of bilateral oophorectomy**									
No	429	83.4	346	88.6	1.00 (ref)	55	85.9	1.00 (ref)	0.42
Yes	85	16.6	45	11.4	**0.59 (0.38, 0.90)**	9	14.1	0.82 (0.37, 1.80)	
*P*-value					**0.015**			0.62	
**BBD histology**									
Normal/non-proliferative	384	74.7	243	62.2	1.00 (ref)	43	67.2	1.00 (ref)	0.45
Proliferative without atypia	124	24.1	127	32.5	**1.70 (1.25, 2.30)**	20	31.3	1.49 (0.84, 2.67)	
Proliferative with atypia	6	1.2	21	5.4	**5.48 (2.14, 14.01)**	1	1.6	–	
*P*-trend					**< 0.0001**			–	
**Subjective impression of involution**									
None/mildly involuted (0–24%)	235	45.7	187	47.8	1.00 (ref)	27	42.2	1.00 (ref)	0.85
Partially involuted (25–74%)	75	14.6	78	20.0	1.38 (0.93, 2.04)	12	18.8	1.48 (0.70, 3.13)	
Completely involuted (≥ 75%)	135	26.3	84	21.5	0.87 (0.61, 1.24)	15	23.4	1.07 (0.53, 2.13)	
No TDLU observed	69	13.4	42	10.7	0.69 (0.43, 1.10)	10	15.6	1.28 (0.57, 2.91)	
*P*-trend^b^					0.68			0.90	
**Columnar cell lesions**^**c**^									
None	450	87.9	328	84.1	1.00 (ref)	53	82.8	1.00 (ref)	0.94
Present with/without atypia	62	12.1	62	15.9	**1.54 (1.03, 2.29)**	11	17.2	1.50 (0.73, 3.07)	
*P*-value					**0.034**			0.27	

Fifty-nine cases who had missing ER status were excluded from modeling analyses. *BBD* benign breast disease, *CI* confidence interval, *ER* estrogen receptor, *OR* odds ratio. ^a^Averaged frequencies and percentages. ^b^Women with zero-TDLU observed were not included in trend tests or heterogeneity tests. ^c^Two controls and two cases were missing for columnar cell lesions. *OR and 95% CI estimates were calculated using polytomous logistic regression models adjusted for categorized BBD diagnosis calendar year as a trend, continuous age at BBD and follow-up period from BBD diagnosis to breast cancer diagnosis, family history of breast cancer in 1st-degree relatives, history of bilateral oophorectomy, BBD histology, and parity. ^†^*P*-heterogeneity was calculated from case-case analyses. Involution and columnar cell lesion associations were adjusted for all factors above the line indicated in OR*

### Breast cancer risk by grade and tumor size among women with BBD

While breast cancer risk associations for patient and histologic characteristics were generally consistent by tumor grade, we found suggestive evidence that higher levels of involution were inversely associated with reduced risk among well-differentiated tumors (complete vs no involution, OR (95% CI) = 0.51 (0.29, 0.90), Supplemental Table 3); this association was not evident among moderately or poorly differentiated tumors, *P*-het = 0.054. Associations for patient and histologic characteristics by tumor size (< 20 mm indicating small and > 20 mm larger tumors) are presented in Supplemental Table 4. We did not observe any significant heterogeneity by tumor size, although associations of the severity of BBD histology (*P* < 0.0001) and presence of CCLs (*P* = 0.038) with increased breast cancer risk were somewhat stronger among patients with smaller tumors.

### Breast cancer risk by menopausal status at BBD diagnosis and before and after 1993

We performed additional analyses stratified by menopausal status using factors that have been significantly associated with breast cancer risk, in our study population (Table 3). The association of CCLs with elevated breast cancer risk was most apparent for postmenopausal women (OR = 2.08, 95% CI = 1.21, 3.58), *P*-het = 0.09.

**Table 3:** Associations between key risk factors and breast cancer risk stratified by menopausal status at BBD diagnosis (averaged frequencies)

Variable	Premenopausal women, ***N*** = 449^**a**^					Postmenopausal women, ***N*** = 579^**a**^					***P-***het^**†**^
	Control ***N*** = 217		Case ***N*** = 233		Multivariable model*	Control ***N*** = 297		Case ***N*** = 282		Multivariable model*	
	***N***^**a**^	%^**a**^	***N***^**a**^	%^**a**^	OR (95% CI)*	***N***^**a**^	%^**a**^	***N***^**a**^	%^**a**^	OR (95% CI)*	
**Age at first full-term birth/years**											
Nulliparous/≥ 30	57	26.3	83	35.7	1.00 (ref)	50	17.0	56	20.0	1.00 (ref)	0.35
< 30	160	73.7	150	64.3	**0.59 (0.38, 0.90)**	247	83.0	226	80.0	0.81 (0.47, 1.38)	
*P*-value					**0.015**					0.43	
**Family history of breast cancer**											
No	190	87.5	186	80.0	1.00 (ref)	244	82.2	225	79.9	1.00 (ref)	0.28
Yes	27	12.5	47	20.0	1.75 (1.00, 3.07)	53	17.8	57	20.1	1.18 (0.76, 1.83)	
*P*-value					0.052					0.46	
**History of bilateral oophorectomy**											
No	–	–	–	–	–	212	71.3	221	78.3	1.00 (ref)	–
Yes	–	–	–	–	–	85	28.7	61	21.7	**0.66 (0.44, 0.99)**	
*P*-value					–					**0.044**	
**BBD histology**											
Normal/non-proliferative	178	81.9	157	67.5	1.00 (ref)	206	69.5	167	59.4	1.00 (ref)	0.47
Proliferative without atypia	37	17.2	67	28.7	**2.06 (1.27, 3.33)**	87	29.2	97	34.2	1.41 (0.97, 2.03)	
Proliferative with atypia	2	0.9	9	3.9	**5.45 (1.14, 26.15)**	4	1.4	18	6.4	**5.65 (1.86, 17.14)**	
*P*-trend					**0.015**					0.41	
**Columnar cell lesions**^**b**^											
None	179	83.2	188	81.1	1.00 (ref)	271	91.3	238	84.6	1.00 (ref)	0.09
Present with/without atypia	36	16.9	44	18.9	1.09 (0.65, 1.82)	26	8.7	43	15.4	**2.08 (1.21, 3.58)**	
*P*-value					0.73					**0.008**	

*BBD* benign breast disease, *CI* confidence interval, *OR* odds ratio. ^a^Averaged frequencies. ^b^Two controls (all premenopausal women) and two cases (1 pre- and 1 postmenopausal women) were missing for columnar cell lesions. *OR and 95% CI estimates were calculated using unconditional logistic regression models adjusted for continuous age at BBD and follow-up period from BBD diagnosis to breast cancer diagnosis, categorized BBD calendar year as a trend, family history of breast cancer in 1st-degree relatives, BBD histology, and parity. Postmenopausal women were additionally adjusted for history of bilateral oophorectomy. ^†^*P*-heterogeneity was calculated from multivariable analyses comparing pre- versus postmenopausal women

We did not find evidence of heterogeneity in risk factor associations before and after 1993 when 75% of women over 45 had had a screening mammogram (Supplemental Table 5), suggesting associations are robust.

## Discussion

There are few BBD cohort studies with comprehensive follow-up and detailed characteristics on subsequently diagnosed invasive tumors. Among a large, well-characterized cohort of patients diagnosed with BBD, we expanded upon previous analyses in this population [7, 8] that evaluated associations of well-established breast cancer risk factors and BBD features with breast cancer risk to determine herein if there exists possible etiologic heterogeneity using tumor characteristic data obtained from the long-standing high-quality Kaiser tumor cancer registry [16]. Determining risk associations by tumor subtypes has been increasingly recognized as an important area of research [20, 21]. We comprehensively analyzed histopathologic features of the BBD biopsy and clinicopathologic characteristics of the breast tumors that developed subsequent to BBD diagnosis. We found that most breast cancers diagnosed among BBD patients within this general community healthcare plan were the low-stage, ER-positive tumors that tend to be highly responsive to treatments. Compared with patients with non-proliferative BBD, those with proliferative BBD with atypia had an over fivefold increased risk of ER-positive breast cancer. Our analyses provided limited evidence for heterogeneity in risk factor associations that we evaluated by tumor characteristics. Importantly, histology and presence of CCLs at the time of BBD were independently associated with subsequent breast cancer risk irrespective of ER status, or tumor size or grade. These data provide further support for CCLs as a breast cancer risk factor, especially for postmenopausal BBD patients.

### Patient characteristics and breast cancer risk

We extended prior findings by evaluating etiologic heterogeneity for risk factors thought to be most relevant for subsequent risk among BBD patients. Consistent with multiple studies of women diagnosed with sporadic breast cancer [9–12] and with the Mayo Clinic BBD cohort [6], we observed that parity/age at first birth was significantly associated with future breast cancer risk. Also consistent with limited data from other BBD cohorts [6], we found that history of bilateral oophorectomy was associated with reduced breast cancer risk, whereas a positive family history of breast cancer in a 1st-degree relative tended to be associated with increased breast cancer risk. We were able to further evaluate potential etiologic heterogeneity in risk associations, because our BBD study is nested within a single, large, well-defined population with access to health care and with a long-standing tumor tissue registry that has collected data since the 1960s. Although the number of ER-negative tumors was limited, we confirmed patterns of association by ER status that have also been observed among women diagnosed with sporadic breast cancers [9–12]. For example, early age at first birth showed an inverse association with ER-positive tumors suggesting that reproductive risk factor associations among BBD patients may be similar to those observed in the general population.

### ADH

It has long been established that ADH is a high-risk precursor lesion, conferring a 4–5-fold increase in the risk of breast cancer development [3]. Indeed, our analysis of BBD patients showed that compared to non-proliferative lesions, a diagnosis of ADH was associated with over 5-fold increased breast cancer risk; however, this association was limited to the risk of ER-positive breast cancer, with little or no risk observed for ER-negative disease. Limited data from cohorts have reported on the relation of ADH by hormone receptor subtype. Consistent with our results, a previous population-based case-control study, CASH, found that history of any benign breast disease was associated only with increased risk of ER-positive luminal A tumors (OR = 1.89, 95% CI 1.43–2.50) [22]. These findings support the hypothesis that benign lesions are more likely to be hormone receptor positive with less genomic instability [23–26]. Moreover, the finding that ADH might be more relevant for ER-positive breast cancer risk is also consistent with hormonal chemoprevention trials which show a significant reduction in risk in women diagnosed with ADH [5, 27].

### Proliferative BBD without atypia

Proliferative disease without atypia is a conglomerate of multiple different pathologies. Radial scars are proliferative lesions that visually appear similar to tumors on mammograms. Pathologically, they are associated with epithelial elements and/or other proliferative lesions such as sclerosing adenosis. Recent analyses in two Swedish cohorts recruited through mammography screening programs from 2001 to 2013 with over 75,000 subjects did not find any significant heterogeneity when evaluating the associations of non-proliferative or proliferative BBD lesions with risk of molecularly defined subtypes of breast cancer; however, a limitation in this study was that there was no separation of proliferative lesions with or without atypia [28]. Our analysis using the Dupont and Page classification of BBD histology showed that PDWA was associated with increased risk of both ER+ and ER− disease. This is consistent with the hypothesis that these lesions serve as precursor lesions in the natural history of breast cancer [3]. The Nurses’ Health Study and the Mayo BBD study showed radial scars to be one of the PDWA lesions associated with breast cancer risk, with an almost 2-fold increased risk [6, 29]. The Nurses’ Health Study cohorts showed an independent association after adjusting for BBD histology. After restricting analyses to BBD patients with proliferative disease, we did not find a significant increase in risk associated with sclerosing adenosis or radial scar. Reasons for these discrepancies could be differences in the study populations as the calendar periods for all three of the cohorts overlap; the Nurses BBD cohort were women who reported a first diagnosis of BBD between 1976 and 1998; the Mayo BBD cohort is a hospital-based referral center that may see more high-risk women but with a similar calendar period to our study with BBD diagnoses occurring between 1967 and 2001. As our study was embedded in a healthcare organization where the women are actively followed and had access to mammography screening, breast cancers detected in our study may be found earlier than in other cohorts which makes direct comparisons difficult.

### Columnar cell lesions

While its generally accepted that ADH, lobular neoplasia, and ductal carcinoma in situ (DCIS) are precursor lesions for breast cancer [30, 31], evidence is accumulating that CCLs may also be a precursor, although conveying lower risk particularly among populations with access to mammography screening [32–34]. Our data support CCLs as a common risk factor for both ER-positive and ER-negative breast cancers with relative risk estimates of around 1.5 after accounting for BBD histology, for both tumor types. Molecular analyses of CCLs suggest that some alterations occur early and mimic those observed in coincident established precursor lesions such as DCIS [3, 8, 23, 33, 35–42]. There are three other studies that have evaluated CCLs in BBD cohorts, Nashville, Nurses, and Mayo, and our data are consistent with the findings of all three [23, 35, 36, 40]. Only the Nurses BBD cohort obtained tumor characteristics on cases and also did not find any significant differences by ER status or grade, which is consistent with our data. Current management of CCL, according to the Mayo Clinic review, suggests that these patients should be managed with annual clinical breast exams and mammography [3]. Our data suggest CCL might be a risk factor for both ER+ and ER− disease, which has not been observed previously and requires further investigation.

### Involution status

Reduced lobular involution has been previously shown to be a significant factor associated with elevated breast cancer risk among women with BBD [19, 43, 44]. In the present study, we found that involution was weakly inversely related with breast cancer risk for ER+ disease. Further, higher levels of lobular involution were inversely and significantly associated with risk for well-differentiated tumors, a finding which was not observed for moderately or poorly differentiated tumors. As there were relatively few hormone-negative cases in this population, we had limited power to test for differences by ER status. A limitation of this study is the absence of data on menopausal hormone therapy (MHT) use after BBD diagnoses, as MHT uptake was rising at the same time that mammography screening was increasingly being adopted [16]. Given previous studies showing that recent MHT use reduces involution levels among current but not former users [45], we hypothesize that MHT use post-BBD diagnosis may be an unmeasured negative confounder attenuating involution associations towards the null. Overall, it is unclear whether screening practices, unmeasured MHT use, or/and both might confound observed associations with involution. Additional contemporary studies in other populations with managed health care might help clarify the relationship of involution with future breast cancer risk.

This is a unique cohort of women enrolled in a general community healthcare plan, providing the population access to screening and preventative services that may not be typical for other subsets of the US population. It is not currently known whether women with BBD are followed and screened more closely compared to women without BBD diagnoses. Current data from a study of over 42,000 screened women in Spain found that women with a previous benign breast disease diagnosis had a higher cumulative risk of screen-detected cancer and interval cancers, consistent with data supporting BBD as a risk factor for breast cancer regardless of the mode of detection [46]. Whether women with BBD in our cohort are more likely than the general population to participate in screening is not known but could be the subject of future research.

### Strengths/limitations

A limitation of our study is that risk estimates are based on a BBD patient population diagnosed on excisional biopsies during a calendar period spanning the adoption of widespread mammography screening, which became more commonplace in KPNW around 1993; thus, associations may not be reflective of BBD diagnosed in more recent years. Since 1995, advances in breast imaging technologies have resulted in a shift in diagnostic biopsy procedures to core needle biopsies, which currently comprise about 80% of biopsies in the USA [47]. Based on data from the US Breast Cancer Surveillance Consortium, risks associated with high-risk ADH lesions on excisional biopsy were lower when diagnosed via core biopsy (6.7% vs 5.0%), perhaps reflecting the size of the ADH focus [47]. Another limitation as noted earlier was the absence of risk factor data after BBD diagnosis and in particular on MHT use, which has been noted to be prevalent at KPNW at this time [16] and could have biased the results, especially with respect to the findings for involution. The small number of patients with ER-negative breast cancers is another limitation. Our study had multiple strengths: it was a nested case-control study embedded within a large, well-characterized BBD cohort with lengthy follow-up and access to archival BBD tissues and well-established detailed tumor registry data, the latter aspect allowing us to provide one of the most detailed analyses to date of tumor characteristics of breast cancers diagnosed among the vast majority of patients. Moreover, because we studied women in a healthcare management organization, we could also evaluate temporal changes in access to mammography screening, data that are limited in other cohorts.

## Summary and conclusions

Within this BBD cohort, the largest to date with longitudinal follow-up, we provide breast cancer risk factor associations within an HMO patient population [7]. Our data provide further evidence that PDWA is associated with both ER-positive and ER-negative disease. Further, we show CCLs are associated with moderate increases in breast cancer risk, independent of BBD histology, and irrespective of ER status, in agreement with previous studies. Given the predominance of low-stage ER-positive tumors that developed among this cohort, our findings suggest that invasive cancers that develop subsequent to a BBD diagnosis are likely highly treatable with low mortality. Histologic evaluation of BBD biopsies is a promising avenue for the identification of new risk factors for different molecular subtypes of breast cancer and could inform the natural history of disease; however, given complex relationships between screening and diagnosis, along with secular changes in risk factor prevalence, contemporary prospective studies are needed to clarify the relationships of factors that may influence progression of BBD to cancer.

## Supplementary Information

**Additional file 1: **Supplemental methods. **Supplementary Figure 1.** Steps of multiple imputation (for more details see Appendix A). **Supplementary Table 1.** Characteristics of BBD histology and breast cancers among cases by calendar year of BBD and breast cancer diagnoses (*N* = 514). **Supplemental Table 2.** Comparison of associations between select patient characteristics and histologic features with breast cancer risk in conditional and unconditional logistic regression models using multiple imputation dataset (*N* = 1028). **Supplemental Table 3.** Associations between select patient characteristics and histologic features with breast cancer risk by tumor grade (*N* = 922). **Supplemental Table 4.** Associations between select patient characteristics and histologic features with breast cancer risk by tumor size (*N* = 1013). **Supplementary Table 5.** Associations between demographic and histologic features with breast cancer risk by BBD calendar year before vs. after 1993 (N = 1028). **Appendix A.** Distribution of numeric variables before (top) and after (bottom) multiple imputations.

## Data Availability

The datasets generated or analyzed during the current study are not publicly available due to data privacy of patients. The authors will make the data available upon reasonable request and with permission of the Kaiser Permanente Center for Health Research in Portland, Oregon.

## References

[1] Dupont WD, Page DL (1985). Risk factors for breast cancer in women with proliferative breast disease. N Engl J Med.

[2] Allison KH, Abraham LA, Weaver DL, Tosteson AN, Nelson HD, Onega T, Geller BM, Kerlikowske K, Carney PA, Ichikawa LE, Buist DS, Elmore JG. Trends in breast biopsy pathology diagnoses among women undergoing mammography in the United States: a report from the Breast Cancer Surveillance Consortium. Cancer. 2015;121(9):1369–78. 10.1002/cncr.29199. 10.1002/cncr.29199 PMC4419038 25603785

[3] Neal L, Sandhu NP, Hieken TJ, Glazebrook KN, Mac Bride MB, Dilaveri CA, Wahner-Roedler DL, Ghosh K, Visscher DW (2014). Diagnosis and management of benign, atypical, and indeterminate breast lesions detected on core needle biopsy. Mayo Clin Proc.

[4] Hartmann LC, Degnim AC, Santen RJ, Dupont WD, Ghosh K (2015). Atypical hyperplasia of the breast--risk assessment and management options. N Engl J Med.

[5] Cuzick J, Sestak I, Thorat MA (2015). Impact of preventive therapy on the risk of breast cancer among women with benign breast disease. Breast.

[6] Pankratz VS, Degnim AC, Frank RD, Frost MH, Visscher DW, Vierkant RA, Hieken TJ, Ghosh K, Tarabishy Y, Vachon CM, Radisky DC, Hartmann LC (2015). Model for individualized prediction of breast cancer risk after a benign breast biopsy. J Clin Oncol.

[7] Arthur R, Wang Y, Ye K, Glass AG, Ginsberg M, Loudig O, Rohan T (2017). Association between lifestyle, menstrual/reproductive history, and histological factors and risk of breast cancer in women biopsied for benign breast disease. Breast Cancer Res Treat.

[8] Kabat GC, Jones JG, Olson N, Negassa A, Duggan C, Ginsberg M, Kandel RA, Glass AG, Rohan TE (2010). A multi-center prospective cohort study of benign breast disease and risk of subsequent breast cancer. Cancer Causes Control.

[9] Palmer JR, Boggs DA, Wise LA, Ambrosone CB, Adams-Campbell LL, Rosenberg L (2011). Parity and lactation in relation to estrogen receptor negative breast cancer in African American women. Cancer Epidemiol Biomark Prev.

[10] Palmer JR, Viscidi E, Troester MA, et al. Parity, lactation, and breast cancer subtypes in African American women: results from the AMBER Consortium. J Natl Cancer Inst. 2014;106(10):dju237. 10.1093/jnci/dju237. 10.1093/jnci/dju237 PMC4271113 25224496

[11] Sighoko D, Ogundiran T, Ademola A, Adebamowo C, Chen L, Odedina S, Anetor I, Ndom P, Gakwaya A, Ojengbede O, Huo D, Olopade OI. Breast cancer risk after full-term pregnancies among African women from Nigeria, Cameroon, and Uganda. Cancer. 2015;121(13):2237–43. 10.1002/cncr.29305. 10.1002/cncr.29305 PMC4573769 25781581

[12] Yang XR, Chang-Claude J, Goode EL, Couch FJ, Nevanlinna H, Milne RL, Gaudet M, Schmidt MK, Broeks A, Cox A, Fasching PA, Hein R, Spurdle AB, Blows F, Driver K, Flesch-Janys D, Heinz J, Sinn P, Vrieling A, Heikkinen T, Aittomaki K, Heikkila P, Blomqvist C, Lissowska J, Peplonska B, Chanock S, Figueroa J, Brinton L, Hall P, Czene K, Humphreys K, Darabi H, Liu J, Van ‘t Veer LJ, van Leeuwen FE, Andrulis IL, Glendon G, Knight JA, Mulligan AM, O’Malley FP, Weerasooriya N, John EM, Beckmann MW, Hartmann A, Weihbrecht SB, Wachter DL, Jud SM, Loehberg CR, Baglietto L, English DR, Giles GG, CA ML, Severi G, Lambrechts D, Vandorpe T, Weltens C, Paridaens R, Smeets A, Neven P, Wildiers H, Wang X, Olson JE, Cafourek V, Fredericksen Z, Kosel M, Vachon C, Cramp HE, Connley D, Cross SS, Balasubramanian SP, Reed MW, Dork T, Bremer M, Meyer A, Karstens JH, Ay A, Park-Simon TW, Hillemanns P, Arias Perez JI, Menendez Rodriguez P, Zamora P, Benitez J, Ko YD, Fischer HP, Hamann U, Pesch B, Bruning T, Justenhoven C, Brauch H, Eccles DM, Tapper WJ, Gerty SM, Sawyer EJ, Tomlinson IP, Jones A, Kerin M, Miller N, McInerney N, Anton-Culver H, Ziogas A (2011). Associations of breast cancer risk factors with tumor subtypes: a pooled analysis from the Breast Cancer Association Consortium studies. J Natl Cancer Inst.

[13] Hartmann LC, Sellers TA, Frost MH, Lingle WL, Degnim AC, Ghosh K, Vierkant RA, Maloney SD, Pankratz VS, Hillman DW, Suman VJ, Johnson J, Blake C, Tlsty T, Vachon CM, Melton LJ, Visscher DW (2005). Benign breast disease and the risk of breast cancer. N Engl J Med.

[14] Tamimi RM, Rosner B, Colditz GA (2010). Evaluation of a breast cancer risk prediction model expanded to include category of prior benign breast disease lesion. Cancer.

[15] Rothman KGS, Lash T (2008). Modern epidemiology.

[16] Glass AG, Lacey JV, Carreon JD, Hoover RN (2007). Breast cancer incidence, 1980-2006: combined roles of menopausal hormone therapy, screening mammography, and estrogen receptor status. J Natl Cancer Inst.

[17] Ali AM, Dawson SJ, Blows FM, Provenzano E, Ellis IO, Baglietto L, Huntsman D, Caldas C, Pharoah PD (2011). Comparison of methods for handling missing data on immunohistochemical markers in survival analysis of breast cancer. Br J Cancer.

[18] Moons KG, Donders RA, Stijnen T, Harrell FE (2006). Using the outcome for imputation of missing predictor values was preferred. J Clin Epidemiol.

[19] Baer HJ, Collins LC, Connolly JL, Colditz GA, Schnitt SJ, Tamimi RM (2009). Lobule type and subsequent breast cancer risk: results from the Nurses’ Health Studies. Cancer.

[20] Lee A, Mavaddat N, Wilcox AN, Cunningham AP, Carver T, Hartley S, Babb de Villiers C, Izquierdo A, Simard J, Schmidt MK, Walter FM, Chatterjee N, Garcia-Closas M, Tischkowitz M, Pharoah P, Easton DF, Antoniou AC (2019). BOADICEA: a comprehensive breast cancer risk prediction model incorporating genetic and nongenetic risk factors. Genet Med.

[21] Mavaddat N, Rebbeck TR, Lakhani SR, Easton DF, Antoniou AC (2010). Incorporating tumour pathology information into breast cancer risk prediction algorithms. Breast Cancer Res.

[22] Gaudet MM, Press MF, Haile RW, Lynch CF, Glaser SL, Schildkraut J, Gammon MD, Douglas Thompson W, Bernstein JL (2011). Risk factors by molecular subtypes of breast cancer across a population-based study of women 56 years or younger. Breast Cancer Res Treat.

[23] Boulos FI, Dupont WD, Simpson JF, Schuyler PA, Sanders ME, Freudenthal ME, Page DL (2008). Histologic associations and long-term cancer risk in columnar cell lesions of the breast: a retrospective cohort and a nested case-control study. Cancer.

[24] Fitzgibbons PL, Henson DE, Hutter RV (1998). Benign breast changes and the risk for subsequent breast cancer: an update of the 1985 consensus statement. Cancer Committee of the College of American Pathologists. Arch Pathol Lab Med.

[25] Lopez-Garcia MA, Geyer FC, Natrajan R, Kreike B, Mackay A, Grigoriadis A, Reis-Filho JS, Weigelt B (2010). Transcriptomic analysis of tubular carcinomas of the breast reveals similarities and differences with molecular subtype-matched ductal and lobular carcinomas. J Pathol.

[26] Page DL, Dupont WD, Rogers LW, Rados MS (1985). Atypical hyperplastic lesions of the female breast. A long-term follow-up study. Cancer.

[27] Cuzick J, Sestak I, Cawthorn S, Hamed H, Holli K, Howell A, Forbes JF, Investigators I-I (2015). Tamoxifen for prevention of breast cancer: extended long-term follow-up of the IBIS-I breast cancer prevention trial. Lancet Oncol.

[28] Holm J, Eriksson L, Ploner A, Eriksson M, Rantalainen M, Li J, Hall P, Czene K (2017). Assessment of breast cancer risk factors reveals subtype heterogeneity. Cancer Res.

[29] Aroner SA, Collins LC, Connolly JL, Colditz GA, Schnitt SJ, Rosner BA, Hankinson SE, Tamimi RM (2013). Radial scars and subsequent breast cancer risk: results from the Nurses’ Health Studies. Breast Cancer Res Treat.

[30] Allred DC, Mohsin SK, Fuqua SA (2001). Histological and biological evolution of human premalignant breast disease. Endocr Relat Cancer.

[31] van Diest PJ (1999). Ductal carcinoma in situ in breast carcinogenesis. J Pathol.

[32] Lee S, Medina D, Tsimelzon A, Mohsin SK, Mao S, Wu Y, Allred DC (2007). Alterations of gene expression in the development of early hyperplastic precursors of breast cancer. Am J Pathol.

[33] Simpson PT, Gale T, Reis-Filho JS, Jones C, Parry S, Sloane JP, Hanby A, Pinder SE, Lee AH, Humphreys S, Ellis IO, Lakhani SR (2005). Columnar cell lesions of the breast: the missing link in breast cancer progression? A morphological and molecular analysis. Am J Surg Pathol.

[34] Sinn HP (2009). Breast cancer precursors: lessons learned from molecular genetics. J Mol Med (Berl).

[35] Aroner SA, Collins LC, Schnitt SJ, Connolly JL, Colditz GA, Tamimi RM (2010). Columnar cell lesions and subsequent breast cancer risk: a nested case-control study. Breast Cancer Res.

[36] Boulos FI, Dupont WD, Schuyler PA, Sanders ME, Page DL, Fedda FA, Simpson JF (2012). Clinicopathologic characteristics of carcinomas that develop after a biopsy containing columnar cell lesions: evidence against a precursor role. Cancer.

[37] Collins LC (2018). Precursor lesions of the low-grade breast neoplasia pathway. Surg Pathol Clin.

[38] Jung MM, Colditz GA, Collins LC, Schnitt SJ, Connolly JL, Tamimi RM (2011). Lifetime physical activity and the incidence of proliferative benign breast disease. Cancer Causes Control.

[39] Meares AL, Frank RD, Degnim AC, Vierkant RA, Frost MH, Hartmann LC, Winham SJ, Visscher DW (2016). Mucocele-like lesions of the breast: a clinical outcome and histologic analysis of 102 cases. Hum Pathol.

[40] Said SM, Visscher DW, Nassar A, Frank RD, Vierkant RA, Frost MH, Ghosh K, Radisky DC, Hartmann LC, Degnim AC (2015). Flat epithelial atypia and risk of breast cancer: a Mayo cohort study. Cancer.

[41] Verschuur-Maes AH, Moelans CB, de Bruin PC, van Diest PJ (2014). Analysis of gene copy number alterations by multiplex ligation-dependent probe amplification in columnar cell lesions of the breast. Cell Oncol (Dordr).

[42] Verschuur-Maes AH, van Gils CH, van den Bosch MA, De Bruin PC, van Diest PJ (2011). Digital mammography: more microcalcifications, more columnar cell lesions without atypia. Modern Pathol.

[43] Figueroa JD, Pfeiffer RM, Brinton LA, Palakal M, Degnim AC, Radisky D, Hartmann LC, Frost M, Stallings-Mann ML, DP, Visscher D, Sherman ME (2016). Standardized measures of lobular involution and subsequent breast cancer risk among women with benign breast disease. Breast Cancer Res Treat.

[44] Milanese TR, Hartmann LC, Sellers TA, Frost MH, Vierkant RA, Maloney SD, Pankratz VS, Degnim AC, Vachon CM, Reynolds CA, Thompson RA, Melton LJ, Goode EL, Visscher DW (2006). Age-related lobular involution and risk of breast cancer. J Natl Cancer Inst.

[45] Figueroa JD, Pfeiffer RM, Patel DA, Linville L, Brinton LA, Gierach GL, Yang XR, Papathomas D, Visscher D, Mies C, Degnim AC, Anderson WF, Hewitt S, Khodr ZG, Clare SE, Storniolo AM, Sherman ME. Terminal duct lobular unit involution of the normal breast: implications for breast cancer etiology. J Natl Cancer Inst. 2014;106(10). 10.1093/jnci/dju286 PMC4200067 25274491

[46] Roman M, Quintana MJ, Ferrer J, Sala M, Castells X (2017). Cumulative risk of breast cancer screening outcomes according to the presence of previous benign breast disease and family history of breast cancer: supporting personalised screening. Br J Cancer.

[47] Menes TS, Kerlikowske K, Lange J, Jaffer S, Rosenberg R, Miglioretti DL (2017). Subsequent breast cancer risk following diagnosis of atypical ductal hyperplasia on needle biopsy. JAMA Oncol.

