# Supplementary material for: Risk factors for breast cancer development by tumor characteristics among women with benign breast disease
Source: Breast Cancer Res. 2021 Mar 18;23:34. doi: 10.1186/s13058-021-01410-1 (PMC7977564; doi:10.1186/s13058-021-01410-1)
Supplement: Supplementary file 1 — Additional file 1: Supplemental methods. Supplementary Figure 1. Steps of multiple imputation (for more details see Appendix A). Supplementary Table 1. Characteristics of BBD histology and breast cancers among cases by calendar year of BBD and breast cancer diagnoses (N = 514). Supplemental Table 2. Comparison of associations between select patient characteristics and histologic features with breast cancer risk in conditional and unconditional logistic regression models using multiple imputation dataset (N = 1028). Supplemental Table 3. Associations between select patient characteristics and histologic features with breast cancer risk by tumor grade (N = 922). Supplemental Table 4. Associations between select patient characteristics and histologic features with breast cancer risk by tumor size (N = 1013). Supplementary Table 5. Associations between demographic and histologic features with breast cancer risk by BBD calendar year before vs. after 1993 (N = 1028). Appendix A. Distribution of numeric variables before (top) and after (bottom) multiple imputations. [file 13058_2021_1410_MOESM1_ESM.docx]

**Supplemental Methods and Tables:**

Supplemental methods

*Histologic assessment of TDLU involution*

Qualitative measures of TDLU involution were assessed microscopically by a pathologist (MAD) as previously described [1]. Briefly, samples containing one or more normal TDLUs were assessed for the degree of involution classified as none/mildly involuted (0–24%), partially involuted (25–74%), or completely involuted (>75%).

For standardized measures of TDLU involution, stained H&E tissue sections were digitized at 20X using a Hamamatsu NanoZoomer 2.0HT (Hamamatsu, Bridgewater NJ ), and managed for web-based viewing and annotation with Digital Image Hub software (Slidepath/Leica, Dublin, Ireland), as previously described [2]. Only TDLUs that appeared entirely normal or showed focal benign changes (duct dilatation, metaplasia, hyperplasia) were assessed. TDLUs were not considered normal if more than half the acini were dilated 2-3 times the normal diameter or if there were metaplastic changes involving more than half the acini. TDLUs showing ductal hyperplasia, defined as ducts or acini lined by more than a single epithelial cell layer, were not considered normal and therefore not assessed for involution analysis. In addition, the numbers of TDLUs with proliferative changes, and therefore not considered normal or suitable for assessment of involution, were recorded.

Images of sections were reviewed masked to other data to estimate percentage of fat (in deciles), and to enumerate the total number of normal TDLUs (as defined above). Up to ten normal TDLUs were reviewed sequentially to assess: 1) TDLU span, measured with an electronic ruler (microns) and 2) number of acini per TDLU (1: 2-10; 2: 11-20; 3: 21-30; 4: 31-40; 5: 50+,). To determine standardized number of TDLUs, total tissue area was measured using the lasso drawing function in software is called ASAP (version 1.7.2) to outline the perimeter of the tissue (in mm^2^). Prior reports have found that assessment of at least six TDLUs per section per patient provides stable representative measures of TDLU characteristics [3-5]. For samples with multiple tissue pieces, the area was summed across the number of tissues represented on the H&E image. For acini counts/TDLU and TDLU span measures, we used the median of the values obtained across the multiple TDLUs measured for each woman. Proportion of cases and controls that had 6 TDLUs reviewed was 75.9% and 72.8%, respectively.

*Multiple imputation*

Multiple imputation variables that were included were case/control status, BBD calendar year at diagnosis, age at BBD diagnosis, laterality of BBD diagnosis, age at menarche, age at first live birth, number of pregnancies, menopausal status, history of bilateral oophorectomy before BBD biopsy date, family history of breast cancer in first degree relatives, height, weight, hysterectomy before BBD biopsy. In addition, pathology variables number of tissue pieces on slide and total tissue area, percent fat of tissue, whether epithelium present, if normal TDLUs observed, subjective impression of involution, median acini number/span measures, number of normal TDLUs, number of fibrocystic lobules and columnar cell lesion presence and BBD histology (Page criteria classification). For cases additional variables included were age at breast cancer diagnosis, breast cancer calendar year at diagnosis, ICD0 site, ER, PR, HER2, tumour histology, stage, grade, node and size (See Supplemental Figure 1 and Appendix A).

References:

1. Milanese TR, Hartmann LC, Sellers TA, Frost MH, Vierkant RA, Maloney SD, Pankratz VS, Degnim AC, Vachon CM, Reynolds CA, Thompson RA, Melton LJ, 3rd, Goode EL, Visscher DW: **Age-related lobular involution and risk of breast cancer**. *J Natl Cancer Inst* 2006, **98**(22):1600-1607.

2. Figueroa JD, Pfeiffer RM, Patel DA, Linville L, Brinton LA, Gierach GL, Yang XR, Papathomas D, Visscher D, Mies C, Degnim AC, Anderson WF, Hewitt S, Khodr ZG, Clare SE, Storniolo AM, Sherman ME: **Terminal duct lobular unit involution of the normal breast: implications for breast cancer etiology**. *J Natl Cancer Inst* 2014, **106**(10).

3. Vierkant RA, Hartmann LC, Pankratz VS, Anderson SS, Radisky D, Frost MH, Vachon CM, Ghosh K, Distad TJ, Degnim AC, Reynolds CA: **Lobular involution: localized phenomenon or field effect?** *Breast Cancer Res Treat* 2009, **117**(1):193-196.

4. McKian KP, Reynolds CA, Visscher DW, Nassar A, Radisky DC, Vierkant RA, Degnim AC, Boughey JC, Ghosh K, Anderson SS, Minot D, Caudill JL, Vachon CM, Frost MH, Pankratz VS, Hartmann LC: **Novel breast tissue feature strongly associated with risk of breast cancer**. *J Clin Oncol* 2009, **27**(35):5893-5898.

5. Yang XR, Figueroa JD, Falk RT, Zhang H, Pfeiffer RM, Hewitt SM, Lissowska J, Peplonska B, Brinton L, Garcia-Closas M, Sherman ME: **Analysis of Terminal Duct Lobular Unit (TDLU) Involution in Luminal A and Basal Breast Cancers**. *Breast Cancer Res* 2012, **14**(2):R64.

Supplementary Figure 1. Steps of multiple imputation (for more details see Appendix A)

**Multiple imputation**

1) Entered all selected variables into the process.

2) To avoid collinearity in prediction modeling, applied stepwise selection, setting the maximum number of variables to be included as predictors in the regression as 5 and the minimum marginal r^2^ as 0.01.

3) The entire imputation process ran 5 times and generated 5 imputed datasets.

**Data checking and manipulation after multiple imputation**

In each of the 5 imputed datasets, checked distributions of each variable that previously had missing data and compared their distributions before and after the multiple imputations.

**Data preparations before multiple imputation**

1) Selected any key variables related to outcome variables.

2) Calculated percentages of missingness in each selected variable and checked outliers or implausible values in these variables.

4) Assigned the status of bilateral oophorectomy as “No” in premenopausal women whose history of bilateral oophorectomy were missing.

5) Set boundary for each continuous variable and added restrictions with logic statements.

# Supplementary Table 1: Characteristics of BBD histology and breast cancers among cases by calendar year of BBD and breast cancer diagnoses (N=514)

|  | **BBD diagnosis calendar year** | | | | |  | **Breast cancer diagnosis calendar year** | | | | |
| --- | --- | --- | --- | --- | --- | --- | --- | --- | --- | --- | --- |
|  | **Before 1993 (n=322)** | | **1993 and after (n=192)** | |  |  | **Before 1993 (n=93)** | | **1993 and after (n=421)** | |  |
|  | **N** | **%** | **N** | **%** | **Pᵃ** |  | **N** | **%** | **N** | **%** | **Pᵃ** |
| **Age at BBD/years** |  |  |  |  | **<0.0001** |  |  |  |  |  | 0.26 |
| <40 | 81 | 25.2 | 9 | 4.7 |  |  | 21 | 22.6 | 69 | 16.4 |  |
| 40-49 | 90 | 28.0 | 51 | 26.6 |  |  | 22 | 23.7 | 119 | 28.3 |  |
| 50-59 | 82 | 25.5 | 63 | 32.8 |  |  | 20 | 21.5 | 125 | 29.7 |  |
| 60-69 | 47 | 14.6 | 39 | 20.3 |  |  | 19 | 20.4 | 67 | 15.9 |  |
| ≥70 | 22 | 6.8 | 30 | 15.6 |  |  | 11 | 11.8 | 41 | 9.7 |  |
| mean (SD) | 49.8 (12.4) | | 56.32 (11.5) | |  |  | 52.6 (13.4) | | 52.1 (12.3) | |  |
| median (IQR) | 48.4 (39.9, 57.9) | | 55.9 (48.0, 63.5) | |  |  | 52.0 (41.1, 62.8) | | 51.5 (42.9, 60.1) | |  |
| **Year of breast cancer diagnosis** |  |  |  |  | **<0.0001** |  |  |  |  |  |  |
| 1973 - 1990 | 72 | 22.6 | 0 | 0 |  |  | -- | -- | -- | -- |  |
| 1991 - 1995 | 67 | 20.8 | 1 | 0.5 |  |  | -- | -- | -- | -- |  |
| 1996 - 2000 | 71 | 22.1 | 30 | 15.6 |  |  | -- | -- | -- | -- |  |
| 2001 - 2005 | 62 | 19.3 | 54 | 28.1 |  |  | -- | -- | -- | -- |  |
| 2006 - 2010 | 38 | 11.8 | 68 | 35.4 |  |  | -- | -- | -- | -- |  |
| 2011 - 2013 | 12 | 3.7 | 39 | 20.3 |  |  | -- | -- | -- | -- |  |
| **BBD histology** |  |  |  |  | **0.0046** |  |  |  |  |  | 0.18 |
| Normal/Non-proliferative | 220 | 68.3 | 104 | 54.2 |  |  | 66 | 71.0 | 258 | 61.3 |  |
| Proliferative without atypia | 89 | 27.6 | 74 | 38.5 |  |  | 22 | 23.7 | 141 | 33.5 |  |
| Proliferative with atypia | 13 | 4.0 | 14 | 7.3 |  |  | 5 | 5.4 | 22 | 5.2 |  |
| **Years from BBD to breast cancer diagnosis** | |  |  |  | **<0.0001** |  |  |  |  |  | **<0.0001** |
| ≤10 | 130 | 40.4 | 154 | 80.2 |  |  | 76 | 81.7 | 208 | 49.4 |  |
| >10 | 192 | 59.6 | 38 | 19.8 |  |  | 17 | 18.3 | 213 | 50.6 |  |
| mean (SD) | 13.4 (8.4) | | 6.5 (4.2) | |  |  | 6.4 (5.0) | | 11.8 (8.1) | |  |
| median (IQR) | 12.6 (6.3, 18.7) | | 5.7 (3.1, 9.1) | |  |  | 4.9 (2.5, 8.4) | | 10.1 (5.4, 16.6) | |  |
| **Age at breast cancer diagnosis/year** |  |  |  |  | 0.97 |  |  |  |  |  | **0.0002** |
| <50 | 42 | 13.0 | 25 | 13.0 |  |  | 25 | 26.9 | 42 | 9.9 |  |
| 50-59 | 83 | 25.8 | 50 | 26.0 |  |  | 22 | 23.7 | 111 | 26.4 |  |
| 60-69 | 112 | 34.8 | 70 | 36.5 |  |  | 26 | 28.0 | 156 | 37.1 |  |
| ≥ 70 | 85 | 26.6 | 47 | 24.5 |  |  | 20 | 21.5 | 112 | 26.6 |  |
| mean (SD) | 63.0 (11.6) | | 62.8 (11.3) | |  |  | 59.0 (12.5) | | 63.8 (11.1) | |  |
| median (IQR) | 62.7 (55.1, 71.2) | | 62.7 (54.5, 69.8) | |  |  | 59.3 (47.5, 68.3) | | 63.4 (55.9, 71.5) | |  |
| **Tumor size/mm** |  |  |  |  | 0.16 |  |  |  |  |  | **<0.0001** |
| <10 | 99 | 31.6 | 44 | 24.2 |  |  | 44 | 47.8 | 99 | 24.6 |  |
| 10-20 | 125 | 39.9 | 86 | 47.3 |  |  | 32 | 34.8 | 179 | 44.4 |  |
| >20 | 89 | 28.4 | 52 | 28.6 |  |  | 16 | 17.4 | 125 | 31.0 |  |
| Missing | 9 |  | 10 |  |  |  | 1 |  | 18 |  |  |
| **Tumor grade^c^** |  |  |  |  | 0.67 |  |  |  |  |  | **0.0047^b^** |
| Well differentiated | 81 | 36.5 | 60 | 32.3 |  |  | 0 | 0 | 141 | 35.3 |  |
| Moderately differentiated | 84 | 37.8 | 75 | 40.3 |  |  | 2 | 25.0 | 157 | 39.3 |  |
| Poorly differentiated | 57 | 25.7 | 51 | 27.4 |  |  | 6 | 75.0 | 102 | 25.5 |  |
| Not determined | 100 |  | 6 |  |  |  | 85 |  | 21 |  |  |
| **ER** |  |  |  |  | 0.51 |  |  |  |  |  | 0.59 |
| Negative | 35 | 13.2 | 29 | 15.3 |  |  | 5 | 11.4 | 59 | 14.4 |  |
| Positive | 231 | 86.8 | 160 | 84.7 |  |  | 39 | 88.6 | 352 | 85.6 |  |
| Missing/Unknown | 56 |  | 3 |  |  |  | 49 |  | 10 |  |  |
| **PR** |  |  |  |  | 0.64 |  |  |  |  |  | 0.81 |
| Negative | 79 | 29.7 | 52 | 27.7 |  |  | 12 | 27.3 | 119 | 29.0 |  |
| Positive | 187 | 70.3 | 136 | 72.3 |  |  | 32 | 72.7 | 291 | 71.0 |  |
| Missing/Unknown | 56 |  | 4 |  |  |  | 49 |  | 11 |  |  |
| **HER2^d^** |  |  |  |  | **0.028** |  |  |  |  |  | 0.46 |
| Negative | 96 | 73.9 | 128 | 84.2 |  |  | 2 | 66.7 | 222 | 79.6 |  |
| Positive | 30 | 23.1 | 20 | 13.2 |  |  | 1 | 33.3 | 49 | 17.6 |  |
| Equivocal | 4 | 3.1 | 4 | 2.6 |  |  | 0 | 0 | 8 | 2.9 |  |
| Missing/Unknown | 192 |  | 40 |  |  |  | 90 |  | 142 |  |  |
| **Regional lymph nodes** |  |  |  |  | 0.25 |  |  |  |  |  | 0.28 |
| Negative | 203 | 71.7 | 134 | 76.6 |  |  | 55 | 68.8 | 282 | 74.6 |  |
| Positive | 80 | 28.3 | 41 | 23.4 |  |  | 25 | 31.3 | 96 | 25.4 |  |
| Missing | 39 |  | 17 |  |  |  | 13 |  | 42 |  |  |
| **Tumor histology** |  |  |  |  | 0.99 |  |  |  |  |  | 0.16^b^ |
| Ductal | 270 | 83.9 | 159 | 82.8 |  |  | 84 | 90.3 | 345 | 82.0 |  |
| Lobular | 29 | 9.0 | 19 | 9.9 |  |  | 7 | 7.5 | 41 | 9.7 |  |
| Mixed ductal/lobular | 16 | 5.0 | 10 | 5.2 |  |  | 1 | 1.1 | 25 | 5.9 |  |
| Other | 7 | 2.2 | 4 | 2.1 |  |  | 1 | 1.1 | 10 | 2.4 |  |
| **Tumor stage** |  |  |  |  | 0.58 |  |  |  |  |  | 0.32^b^ |
| I | 146 | 55.7 | 116 | 61.1 |  |  | 17 | 47.4 | 245 | 59.0 |  |
| II | 91 | 34.7 | 59 | 31.1 |  |  | 17 | 44.7 | 133 | 32.1 |  |
| III | 17 | 6.5 | 12 | 6.3 |  |  | 2 | 5.3 | 27 | 6.5 |  |
| IV | 8 | 3.1 | 3 | 1.6 |  |  | 1 | 2.6 | 10 | 2.4 |  |
| Missing | 60 |  | 2 |  |  |  | 56 |  | 6 |  |  |
| **Subjective impression of involution^e^** |  |  |  |  | 0.079 |  |  |  |  |  | **0.012** |
| None/mildly involuted (0-24%) | 151 | 46.9 | 89 | 46.4 |  |  | 34 | 36.6 | 206 | 48.9 |  |
| Partially involuted (25-74%) | 68 | 21.1 | 33 | 17.2 |  |  | 22 | 23.7 | 79 | 18.8 |  |
| Completely involuted (≥75%) | 89 | 27.6 | 30 | 15.6 |  |  | 32 | 34.4 | 87 | 20.7 |  |
| No TDLU observed | 14 | 4.4 | 40 | 20.8 |  |  | 5 | 5.4 | 49 | 11.6 |  |

Note: Sixty-three percent of cases received their BBD diagnoses before 1993 and 37% of cases in 1993 or after, 18% of cases had their breast cancer diagnoses before 1993 and 82% of cases in 1993 or after. ᵃP values from Chis-square test except where noted; missing data were excluded from analysis; p values less than 0.05 are in bold font. ᵇP values from Fisher exact test. ^c^Patients with tumor grade as "Not determined" were excluded from analysis. ^d^Patients with equivocal HER2 were excluded from analysis. ^e^Patients without TDLU observed were excluded from analysis. BBD, benign breast disease; ER, estrogen receptor status; HER2, human epidermal growth factor; IQR, inter-quartile range; PR, progesterone receptor status; SD, standard deviation.

.

# Supplemental Table 2. Comparison of associations between select patient characteristics and histologic features with breast cancer risk in conditional and unconditional logistic regression models using multiple imputation dataset (N=1028)

|  | **Control (N=514)** | | **Case (N=514)** | | **Multivariable models** | |
| --- | --- | --- | --- | --- | --- | --- |
| **Variable** | **Nᵃ** | **%ᵃ** | **Nᵃ** | **%ᵃ** | **OR (95% CI)*** | **OR (95% CI)†** |
| **Age at first full-term birth/years** |  |  |  |  |  |  |
| Nulliparous/≥30 | 108 | 20.9 | 139 | 27.1 | 1.00 (Ref) | 1.00 (Ref) |
| < 30 | 406 | 79.1 | 375 | 72.9 | **0.71 (0.51, 0.98)** | **0.68 (0.48, 0.95)** |
| P-value |  |  |  |  | **0.036** | **0.023** |
| **Family history of breast cancer** |  |  |  |  |  |  |
| No | 434 | 84.4 | 411 | 80.0 | 1.00 (Ref) | 1.00 (Ref) |
| Yes | 80 | 15.6 | 103 | 20.0 | 1.39 (0.98, 1.96) | 1.36 (0.96, 1.93) |
| P-value |  |  |  |  | 0.064 | 0.084 |
| **History of bilateral oophorectomy** |  |  |  |  |  |  |
| No | 429 | 83.4 | 453 | 88.1 | 1.00 (Ref) | 1.00 (Ref) |
| Yes | 85 | 16.6 | 61 | 11.9 | **0.64 (0.43, 0.95)** | 0.70 (0.47, 1.04) |
| P-value |  |  |  |  | **0.025** | 0.074 |
| **BBD histology** |  |  |  |  |  |  |
| Normal/Non-proliferative | 384 | 74.7 | 324 | 63.0 | 1.00 (Ref) | 1.00 (Ref) |
| Proliferative without atypia | 124 | 24.1 | 163 | 31.7 | **1.65 (1.24, 2.19)** | **1.60 (1.20, 2.12)** |
| Proliferative with atypia | 6 | 1.2 | 27 | 5.3 | **5.56 (2.25, 13.74)** | **5.90 (2.21, 15.73)** |
| P-trend |  |  |  |  | **<0.0001** | **<0.0001** |
|  |  |  |  |  |  |  |
| **Subjective impression of involution** |  |  |  |  |  |  |
| None/mildly involuted (0-24%) | 235 | 45.7 | 240 | 46.7 | 1.00 (Ref) | 1.00 (Ref) |
| Partially involuted (25-74%) | 75 | 14.6 | 101 | 19.7 | 1.33 (0.93, 1.91) | 1.35 (0.93, 1.97) |
| Completely involuted (≥75%) | 135 | 26.3 | 119 | 23.2 | 0.89 (0.65, 1.24) | 0.87 (0.61, 1.24) |
| No TDLU observed | 69 | 13.4 | 54 | 10.5 | 0.74 (0.48, 1.14) | 0.71 (0.46, 1.10) |
| P-trendᵇ |  |  |  |  | 0.65 | 0.62 |
| **Columnar cell lesions^c^** |  |  |  |  |  |  |
| None | 450 | 87.9 | 425 | 83.0 | 1.00 (Ref) | 1.00 (Ref) |
| Present with/without atypia | 62 | 12.1 | 87 | 17.0 | **1.48 (1.03, 2.13)** | **1.49 (1.00, 2.22)** |
| P-value |  |  |  |  | **0.034** | **0.048** |

ᵃAveraged frequencies and percentages. ᵇWomen with zero-TDLU observed were not included in Trend tests. ^c^Two controls and two cases were missing for columnar cell lesions. *OR and 95% CI estimates were calculated using unconditional logistic regression models adjusted for categorized BBD diagnosis calendar year as a trend, continuous age at BBD and follow-up period from BBD diagnosis to breast cancer diagnosis, family history of breast cancer in 1^st^ degree relatives, history of bilateral oophorectomy, BBD histology, and parity. †OR and 95% CI estimates were calculated using conditional logistic regression models adjusted for family history of breast cancer in 1st degree relatives, history of bilateral oophorectomy, BBD histology, and parity. BBD, benign breast disease; CI, confidence interval; ER, estrogen receptor; OR, odds ratio.

# Supplemental Table 3. Associations between select patient characteristics and histologic features with breast cancer risk by tumor grade (N=922)

|  | **Tumor Differentiation Grade** | | | | | | | | | | | |
| --- | --- | --- | --- | --- | --- | --- | --- | --- | --- | --- | --- | --- |
|  | **Control (N=514)** | | **Case, Well (N=141)** | | **Well vs. Control** | **Case, Moderate (N=159)** | | **Moderate vs. Control** | **Case, Poor (N=108)** | | **Poor vs. Control** |  |
| **Variable** | **Nᵃ** | **%ᵃ** | **Nᵃ** | **%ᵃ** | **OR (95% CI)*** | **Nᵃ** | **%ᵃ** | **OR (95% CI)*** | **Nᵃ** | **%ᵃ** | **OR (95% CI)*** | ***P-*het†** |
| **Age at first full-term birth/years** |  |  |  |  |  |  |  |  |  |  |  |  |
| Nulliparous/≥30 | 108 | 20.9 | 35 | 25.0 | 1.00 (Ref) | 41 | 26.0 | 1.00 (Ref) | 29 | 26.7 | 1.00 (Ref) | 0.94 |
| < 30 | 406 | 79.1 | 106 | 75.0 | 0.83 (0.52, 1.32) | 118 | 74.0 | 0.76 (0.48, 1.18) | 79 | 73.3 | 0.77 (0.43, 1.38) |  |
| P-value |  |  |  |  | 0.44 |  |  | 0.22 |  |  | 0.37 |  |
| **Family history of breast cancer** |  |  |  |  |  |  |  |  |  |  |  |  |
| No | 434 | 84.4 | 116 | 82.3 | 1.00 (Ref) | 122 | 77.0 | 1.00 (Ref) | 90 | 83.7 | 1.00 (Ref) | 0.40 |
| Yes | 80 | 15.6 | 25 | 17.7 | 1.16 (0.69, 1.96) | 37 | 23.0 | **1.60 (1.00, 2.56)** | 18 | 16.3 | 1.08 (0.58, 2.01) |  |
| P-value |  |  |  |  | 0.58 |  |  | **0.048** |  |  | 0.81 |  |
| **History of bilateral oophorectomy** |  |  |  |  |  |  |  |  |  |  |  |  |
| No | 429 | 83.4 | 128 | 90.5 | 1.00 (Ref) | 141 | 88.4 | 1.00 (Ref) | 92 | 85.6 | 1.00 (Ref) | 0.30 |
| Yes | 85 | 16.6 | 13 | 9.5 | **0.48 (0.25, 0.92)** | 18 | 11.6 | 0.57 (0.31, 1.06) | 16 | 14.4 | 0.92 (0.48, 1.77) |  |
| P-value |  |  |  |  | **0.027** |  |  | 0.074 |  |  | 0.80 |  |
| **BBD histology** |  |  |  |  |  |  |  |  |  |  |  |  |
| Normal/Non-proliferative | 384 | 74.7 | 88 | 62.4 | 1.00 (Ref) | 98 | 61.6 | 1.00 (Ref) | 68 | 63.0 | 1.00 (Ref) | 0.75 |
| Proliferative without atypia | 124 | 24.1 | 44 | 31.2 | **1.62 (1.05, 2.48)** | 54 | 34.0 | **1.74 (1.16, 2.62)** | 37 | 34.3 | **1.83 (1.15, 2.90)** |  |
| Proliferative with atypia | 6 | 1.2 | 9 | 6.4 | **6.52 (2.19, 19.41)** | 7 | 4.4 | **4.48 (1.42, 14.08)** | 3 | 2.8 | 2.87 (0.69, 11.97) |  |
| P-trend |  |  |  |  | **0.0003** |  |  | **0.0006** |  |  | **0.0065** |  |
|  |  |  |  |  |  |  |  |  |  |  |  |  |
| **Subjective impression of involution** |  |  |  |  |  |  |  |  |  |  |  |  |
| None/mildly involuted (0-24%) | 235 | 45.7 | 77 | 54.6 | 1.00 (Ref) | 76 | 47.8 | 1.00 (Ref) | 45 | 41.7 | 1.00 (Ref) | 0.054 |
| Partially involuted (25-74%) | 75 | 14.6 | 25 | 17.7 | 1.11 (0.65, 1.91) | 26 | 16.4 | 1.14 (0.66, 1.96) | 26 | 24.1 | **2.06 (1.17, 3.63)** |  |
| Completely involuted (≥75%) | 135 | 26.3 | 20 | 14.2 | **0.51 (0.29, 0.90)** | 41 | 25.8 | 1.07 (0.67, 1.71) | 23 | 21.3 | 1.08 (0.61, 1.90) |  |
| No TDLU observed | 69 | 13.4 | 19 | 13.5 | 0.77 (0.41, 1.42) | 16 | 10.1 | 0.60 (0.31, 1.14) | 14 | 13.0 | 1.07 (0.53, 2.14) |  |
| P-trendᵇ |  |  |  |  | **0.043** |  |  | 0.72 |  |  | 0.64 |  |
| **Columnar cell lesions^c^** |  |  |  |  |  |  |  |  |  |  |  |  |
| None | 450 | 87.9 | 114 | 81.4 | 1.00 (Ref) | 140 | 88.1 | 1.00 (Ref) | 92 | 85.2 | 1.00 (Ref) | 0.27 |
| Present with/without atypia | 62 | 12.1 | 26 | 18.6 | **1.99 (1.17, 3.39)** | 19 | 12.0 | 1.21 (0.68, 2.14) | 16 | 14.8 | 1.29 (0.70, 2.38) |  |
| P-value |  |  |  |  | **0.011** |  |  | 0.52 |  |  | 0.42 |  |

106 cases who had "Unknown" tumor grade were excluded from modeling analyses. ᵃAveraged frequencies and percentages. ᵇWomen with zero-TDLU observed were not included in Trend tests or Heterogeneity tests. ^c^Two controls and two cases were missing for columnar cell lesions. *OR and 95% CI estimates were calculated using polytomous logistic regression models adjusted for categorized BBD diagnosis calendar year as a trend, continuous age at BBD and follow-up period from BBD diagnosis to breast cancer diagnosis, family history of breast cancer in 1^st^ degree relatives, history of bilateral oophorectomy, BBD histology, and parity. †P-heterogeneity were calculated from case-case analyses. BBD, benign breast disease; CI, confidence interval; OR, odds ratio.

# Supplemental Table 4. Associations between select patient characteristics and histologic features with breast cancer risk by tumor size (N=1013)

|  | **Tumor size** | | | | | | | | |
| --- | --- | --- | --- | --- | --- | --- | --- | --- | --- |
|  | **Control  (N=514)** | | **Cases, ≤20mm (N=354)** | | **≤20mm vs. Control** | **Cases, >20mm (N=141)** | | **>20mm vs. Control** |  |
| **Variable** | **Nᵃ** | **%ᵃ** | **Nᵃ** | **%ᵃ** | **OR (95% CI)*** | **Nᵃ** | **%ᵃ** | **OR (95% CI)*** | ***P-*het†** |
| **Age at first full-term birth/years** |  |  |  |  |  |  |  |  |  |
| Nulliparous/≥30 | 108 | 20.9 | 94 | 26.5 | 1.00 (Ref) | 38 | 26.8 | 1.00 (Ref) | 0.88 |
| < 30 | 406 | 79.1 | 260 | 73.5 | 0.72 (0.50, 1.05) | 103 | 73.2 | 0.70 (0.44, 1.09) |  |
| P-value |  |  |  |  | 0.087 |  |  | 0.12 |  |
| **Family history of breast cancer** |  |  |  |  |  |  |  |  |  |
| No | 434 | 84.4 | 284 | 80.3 | 1.00 (Ref) | 113 | 80.0 | 1.00 (Ref) | 0.84 |
| Yes | 80 | 15.6 | 70 | 19.7 | 1.36 (0.93, 1.98) | 28 | 20.0 | 1.43 (0.86, 2.39) |  |
| P-value |  |  |  |  | 0.11 |  |  | 0.17 |  |
| **History of bilateral oophorectomy** |  |  |  |  |  |  |  |  |  |
| No | 429 | 83.4 | 313 | 88.4 | 1.00 (Ref) | 126 | 89.7 | 1.00 (Ref) | 0.72 |
| Yes | 85 | 16.6 | 41 | 11.6 | **0.63 (0.41, 0.97)** | 15 | 10.4 | 0.55 (0.29, 1.07) |  |
| P-value |  |  |  |  | 0.034 |  |  | 0.077 |  |
| **BBD histology** |  |  |  |  |  |  |  |  |  |
| Normal/Non-proliferative | 384 | 74.7 | 228 | 64.4 | 1.00 (Ref) | 86 | 61.0 | 1.00 (Ref) | 0.45 |
| Proliferative without atypia | 124 | 24.1 | 107 | 30.2 | **1.54 (1.12, 2.11)** | 50 | 35.5 | **1.93 (1.27, 2.92)** |  |
| Proliferative with atypia | 6 | 1.2 | 19 | 5.4 | **5.46 (2.13, 14.00)** | 5 | 3.6 | **4.04 (1.18, 13.80)** |  |
| P-trend |  |  |  |  | **<0.0001** |  |  | **0.0004** |  |
|  |  |  |  |  |  |  |  |  |  |
| **Subjective impression of involution** |  |  |  |  |  |  |  |  |  |
| None/mildly involuted (0-24%) | 235 | 45.7 | 156 | 44.1 | 1.00 (Ref) | 75 | 53.2 | 1.00 (Ref) | 0.27 |
| Partially involuted (25-74%) | 75 | 14.6 | 71 | 20.1 | 1.41 (0.95, 2.10) | 26 | 18.4 | 1.16 (0.68, 1.99) |  |
| Completely involuted (≥75%) | 135 | 26.3 | 90 | 25.4 | 1.01 (0.70, 1.44) | 24 | 17.0 | 0.64 (0.37, 1.09) |  |
| No TDLU observed | 69 | 13.4 | 37 | 10.5 | 0.81 (0.50, 1.31) | 16 | 11.4 | 0.68 (0.36, 1.31) |  |
| P-trendᵇ |  |  |  |  | 0.78 |  |  | 0.11 |  |
| **Columnar cell lesions^c^** |  |  |  |  |  |  |  |  |  |
| None | 450 | 87.9 | 288 | 81.8 | 1.00 (Ref) | 122 | 86.5 | 1.00 (Ref) | 0.55 |
| Present with/without atypia | 62 | 12.1 | 64 | 18.2 | **1.51 (1.02, 2.24)** | 19 | 13.5 | 1.27 (0.72, 2.25) |  |
| P-value |  |  |  |  | **0.038** |  |  | 0.41 |  |

19 cases who had missing tumor size were excluded from modeling analyses. ᵃAveraged frequencies and percentages. ᵇWomen with zero-TDLU observed were not included in Trend tests or Heterogeneity tests. ^c^Two controls and two cases were missing for columnar cell lesions. *OR and 95% CI estimates were calculated using polytomous logistic regression models adjusted for categorized BBD diagnosis calendar year as a trend, continuous age at BBD and follow-up period from BBD diagnosis to breast cancer diagnosis, family history of breast cancer in 1^st^ degree relatives, history of bilateral oophorectomy, BBD histology, and parity. †P-heterogeneity were calculated from case-case analyses. BBD, benign breast disease; CI, confidence interval; OR, odds ratio.

**Supplementary Table 5. Associations between demographic and histologic features with breast cancer risk by BBD calendar year before vs. after 1993 (N=1028)**

|  | **BBD year <1993 (N= 643)** | | | | |  | **BBD year ≥1993 (N= 385)** | | | | |  |
| --- | --- | --- | --- | --- | --- | --- | --- | --- | --- | --- | --- | --- |
|  | **Control (N=321)** | | **Case (N=322)** | | **Multivariable models** |  | **Control (N=193)** | | **Case (N=192)** | | **Multivariable models** |  |
| **Variable** | **Nᵃ** | **%ᵃ** | **Nᵃ** | **%ᵃ** | **OR (95% CI)*** |  | **Nᵃ** | **%ᵃ** | **Nᵃ** | **%ᵃ** | **OR (95% CI)*** | **P-het†** |
| **Age at first full-term birth/years** |  |  |  |  |  |  |  |  |  |  |  | 0.39 |
| Nulliparous/≥30 | 63 | 19.7 | 87 | 27.0 | 1.00 (Ref) |  | 44 | 23.0 | 52 | 27.2 | 1.00 (Ref) |  |
| < 30 | 258 | 80.3 | 235 | 73.0 | **0.64 (0.43, 0.93)** |  | 149 | 77.0 | 140 | 72.8 | 0.84 (0.47, 1.49) |  |
| P-value |  |  |  |  | **0.021** |  |  |  |  |  | 0.55 |  |
| **Family history of breast cancer** |  |  |  |  |  |  |  |  |  |  |  | 0.73 |
| No | 277 | 86.4 | 265 | 82.2 | 1.00 (Ref) |  | 157 | 81.1 | 146 | 76.1 | 1.00 (Ref) |  |
| Yes | 44 | 13.6 | 57 | 17.8 | 1.46 (0.91, 2.36) |  | 36 | 18.9 | 46 | 23.9 | 1.30 (0.78, 2.16) |  |
| P-value |  |  |  |  | 0.12 |  |  |  |  |  | 0.32 |  |
| **History of bilateral oophorectomy** |  |  |  |  |  |  |  |  |  |  |  | 0.49 |
| No | 280 | 87.2 | 290 | 90.0 | 1.00 (Ref) |  | 149 | 77.2 | 163 | 84.9 | 1.00 (Ref) |  |
| Yes | 41 | 12.8 | 32 | 10.0 | 0.72 (0.41, 1.28) |  | 44 | 22.8 | 29 | 15.1 | **0.55 (0.31, 0.98)** |  |
| P-value |  |  |  |  | 0.27 |  |  |  |  |  | **0.044** |  |
| **BBD histology** |  |  |  |  |  |  |  |  |  |  |  | 0.72 |
| Normal/Non-proliferative | 252 | 78.5 | 220 | 68.3 | 1.00 (Ref) |  | 132 | 68.4 | 104 | 54.2 | 1.00 (Ref) |  |
| Proliferative without atypia | 67 | 20.9 | 89 | 27.6 | **1.65 (1.13, 2.40)** |  | 57 | 29.5 | 74 | 38.5 | **1.71 (1.10, 2.67)** |  |
| Proliferative with atypia | 2 | 0.6 | 13 | 4.0 | **8.31 (1.84, 37.61)** |  | 4 | 2.1 | 14 | 7.3 | **4.41 (1.39, 13.95)** |  |
| P-trend |  |  |  |  | **0.0012** |  |  |  |  |  | **0.0053** |  |
|  |  |  |  |  |  |  |  |  |  |  |  |  |
| **Subjective impression of involution** |  |  |  |  |  |  |  |  |  |  |  | 0.45ᵇ |
| None/mildly involuted (0-24%) | 151 | 9.7 | 151 | 46.9 | 1.00 (Ref) |  | 84 | 43.5 | 89 | 46.4 | 1.00 (Ref) |  |
| Partially involuted (25-74%) | 51 | 47.0 | 68 | 21.1 | 1.33 (0.85, 2.08) |  | 24 | 12.4 | 33 | 17.2 | 1.36 (0.72, 2.56) |  |
| Completely involuted (≥75%) | 88 | 15.9 | 89 | 27.6 | 1.00 (0.67, 1.50) |  | 47 | 24.4 | 30 | 15.6 | 0.65 (0.36, 1.16) |  |
| No TDLU observed | 31 | 27.4 | 14 | 4.4 | 0.45 (0.22, 0.89) |  | 38 | 19.7 | 40 | 20.8 | 1.00 (0.55, 1.80) |  |
| P-trendᵇ |  |  |  |  | 0.86 |  |  |  |  |  | 0.20 |  |
| **Columnar cell lesions^c^** |  |  |  |  |  |  |  |  |  |  |  | 0.76 |
| None | 272 | 85.3 | 258 | 80.4 | 1.00 (Ref) |  | 178 | 92.2 | 167 | 87.4 | 1.00 (Ref) |  |
| Present with/without atypia | 47 | 14.7 | 63 | 19.6 | 1.41 (0.92, 2.17) |  | 15 | 7.8 | 24 | 12.6 | 1.61 (0.79, 3.29) |  |
| P-value |  |  |  |  | 0.11 |  |  |  |  |  | 0.19 |  |

ᵃAveraged frequencies and percentages. ᵇWomen with zero-TDLU observed were not included in statistical tests. ^c^Two controls and two cases were missing for columnar cell lesions. *OR and 95% CI estimates were calculated using unconditional logistic regression models adjusted for continuous age at BBD and follow-up period from BBD diagnosis to breast cancer diagnosis, family history of breast cancer in 1^st^ degree relatives, history of bilateral oophorectomy, BBD histology, and parity. †P-heterogeneity were calculated comparing associations with risk between women diagnosed with BBD before 1993 versus women diagnosed with BBD in 1993 or after. BBD, benign breast disease; CI, confidence interval; OR, odds ratio.

**Appendix A:**

**Distribution of numeric variables before (top) and after (bottom) multiple imputations**

| **Variables** | **Total N** | **N Missing** | **Mean** | **Standard Deviation** | **Median** | **Minimum** | **Maximum** |
| --- | --- | --- | --- | --- | --- | --- | --- |
| Age at BBD, year | 1027 | 1 | 52.13 | 12.44 | 51.5 | 18.7 | 86.6 |
| Weight (kg) | 1021 | 7 | 71.44 | 16.02 | 68.49 | 36.74 | 141.97 |
| Height (meter) | 929 | 99 | 1.64 | 0.07 | 1.63 | 1.45 | 2.03 |
| BMI (derived, kg/m^2^) | 929 | 99 | 26.67 | 5.89 | 25.42 | 16.36 | 56.33 |
| Age at menarche | 809 | 219 | 12.67 | 1.46 | 13 | 9 | 22 |
| Number of pregnancies (among parous women) | 844 | 6 | 2.64 | 1.37 | 2 | 1 | 10 |
| Age at first live birth (among parous women) | 735 | 115 | 23.64 | 4.88 | 23 | 15 | 42 |
| Numbers of fibrocystic lobules | 1019 | 9 | 5.08 | 8.47 | 2 | 0 | 95 |
| Numbers of lymph node (for cases) | 458 | 56 | 1.24 | 3.59 | 0 | 0 | 28 |
| Tumor size (for cases) | 495 | 19 | 17.65 | 15.95 | 14 | 0 | 120 |

| **Variables** | **Total N** | **N Missing** | **Mean** | **Standard Deviation** | **Median** | **Minimum** | **Maximum** |
| --- | --- | --- | --- | --- | --- | --- | --- |
| Age at BBD, year | 5140 | 0 | 52.14 | 12.43 | 51.5 | 18.7 | 86.6 |
| Weight (kg) | 5140 | 0 | 71.46 | 16.02 | 68.49 | 36.74 | 141.97 |
| Height (meter) | 5140 | 0 | 1.64 | 0.07 | 1.63 | 1.45 | 2.03 |
| BMI (derived, kg/m^2^) | 5140 | 0 | 26.69 | 5.95 | 25.42 | 15.29 | 58.05 |
| Age at menarche | 5140 | 0 | 12.67 | 1.45 | 13 | 9 | 22 |
| Number of pregnancies (among parous women) | 4443 | 0 | 2.65 | 1.39 | 2 | 1 | 10 |
| Age at first live birth (among parous women) | 4443 | 0 | 23.68 | 4.81 | 23 | 15 | 42 |
| Numbers of fibrocystic lobules | 5140 | 0 | 5.12 | 8.47 | 2 | 0 | 95 |
| Numbers of lymph node (for cases) | 2570 | 0 | 1.51 | 3.57 | 0 | 0 | 28 |
| Tumor size (for cases) | 2570 | 0 | 18 | 16.01 | 15 | 0 | 120 |

**Distribution of key categorical variables before (left) and after (right) multiple imputations**

|  | | | |  | |  | |  | |  | |  | |  | |  | |  | |  | |
| --- | --- | --- | --- | --- | --- | --- | --- | --- | --- | --- | --- | --- | --- | --- | --- | --- | --- | --- | --- | --- | --- |
| **Age at BBD (year)** | **Frequency** | | **Percent** | | **Cumulative** | | **Cumulative** | |  | | **Age at BBD (year)** | | **Frequency** | | **Percent** | | **Cumulative** | | **Cumulative** | |  |
|  |  |  |  |  | **Frequency** | | **Percent** | |  | |  |  |  |  |  |  | **Frequency** | | **Percent** | |  |
| **<40** | | 181 | | 17.62 | | 181 | | 17.62 | |  | | **<40** | | 905 | | 17.61 | | 905 | | 17.61 | |
| **40-49** | | 280 | | 27.26 | | 461 | | 44.89 | |  | | **40-49** | | 1400 | | 27.24 | | 2305 | | 44.84 | |
| **50-59** | | 292 | | 28.43 | | 753 | | 73.32 | |  | | **50-59** | | 1462 | | 28.44 | | 3767 | | 73.29 | |
| **60-69** | | 172 | | 16.75 | | 925 | | 90.07 | |  | | **60-69** | | 862 | | 16.77 | | 4629 | | 90.06 | |
| **≥70** | | 102 | | 9.93 | | 1027 | | 100 | |  | | **≥70** | | 511 | | 9.94 | | 5140 | | 100 | |
| **Frequency Missing = 1** | | | | | | | | | |  | |  | |  | |  | |  | |  | |

| **BMI (kg/m2)** | **Frequency** | **Percent** | **Cumulative** | **Cumulative** |  | **BMI (kg/m2)** | **Frequency** | **Percent** | **Cumulative** | **Cumulative** |
| --- | --- | --- | --- | --- | --- | --- | --- | --- | --- | --- |
|  |  |  | **Frequency** | **Percent** |  |  |  |  | **Frequency** | **Percent** |
| **≤ 24.9** | 425 | 45.75 | 425 | 45.75 |  | **≤ 24.9** | 2350 | 45.72 | 2350 | 45.72 |
| **25-29** | 281 | 30.25 | 706 | 76 |  | **25-29** | 1552 | 30.19 | 3902 | 75.91 |
| **≥ 30** | 223 | 24 | 929 | 100 |  | **≥ 30** | 1238 | 24.09 | 5140 | 100 |
| **Frequency Missing = 99** | | | | |  |  |  |  |  |  |

| **Age at menarche (year)** | **Frequency** | **Percent** | **Cumulative** | **Cumulative** |  | **Age at menarche (year)** | **Frequency** | **Percent** | **Cumulative** | **Cumulative** |
| --- | --- | --- | --- | --- | --- | --- | --- | --- | --- | --- |
|  |  |  | **Frequency** | **Percent** |  |  |  |  | **Frequency** | **Percent** |
| **≤12** | 368 | 45.49 | 368 | 45.49 |  | **≤12** | 2475 | 48.15 | 2475 | 48.15 |
| **13** | 245 | 30.38 | 613 | 75.77 |  | **13** | 1485 | 28.89 | 3960 | 77.04 |
| **≥14** | 196 | 24.23 | 809 | 100 |  | **≥14** | 1180 | 22.96 | 5140 | 100 |
| **Frequency Missing = 219** | | | | |  |  |  |  |  |  |

***Continued*. Distribution of key categorical variables before (left) and after (right) multiple imputations**

| **Age at first birth (year)** | **Frequency** | **Percent** | **Cumulative** | **Cumulative** |  | **Age at first birth (year)** | **Frequency** | **Percent** | **Cumulative** | **Cumulative** |
| --- | --- | --- | --- | --- | --- | --- | --- | --- | --- | --- |
|  |  |  | **Frequency** | **Percent** |  |  |  |  | **Frequency** | **Percent** |
| **Nulliparous** | 133 | 15.32 | 133 | 15.32 |  | **Nulliparous** | 697 | 13.56 | 697 | 13.56 |
| **<25** | 478 | 55.07 | 611 | 70.39 |  | **<25** | 2876 | 55.95 | 3573 | 69.51 |
| **25-29** | 165 | 19.01 | 776 | 89.4 |  | **25-29** | 1030 | 20.04 | 4603 | 89.55 |
| **≥30** | 92 | 10.6 | 868 | 100 |  | **≥30** | 537 | 10.45 | 5140 | 100 |
| **Frequency Missing = 160** | | | | |  |  |  |  |  |  |
|  | |  |  |  |  |  |  |  |  |  |
| **Number of pregnancies** | **Frequency** | **Percent** | **Cumulative** | **Cumulative** |  | **Number of pregnancies** | **Frequency** | **Percent** | **Cumulative** | **Cumulative** |
|  |  |  | **Frequency** | **Percent** |  |  |  |  | **Frequency** | **Percent** |
| **Nulliparous** | 132 | 13.52 | 132 | 13.52 |  | **Nulliparous** | 697 | 13.56 | 697 | 13.56 |
| **1** | 135 | 13.83 | 267 | 27.36 |  | **1** | 706 | 13.74 | 1403 | 27.3 |
| **2** | 337 | 34.53 | 604 | 61.89 |  | **2** | 1764 | 34.32 | 3167 | 61.61 |
| **3** | 203 | 20.8 | 807 | 82.68 |  | **3** | 1079 | 20.99 | 4246 | 82.61 |
| **≥4** | 169 | 17.32 | 976 | 100 |  | **≥4** | 894 | 17.39 | 5140 | 100 |
| **Frequency Missing = 52** | | | | |  |  |  |  |  |  |
|  |  |  |  |  |  |  |  |  |  |  |
| **Parity** | **Frequency** | **Percent** | **Cumulative** | **Cumulative** |  | **Parity** | **Frequency** | **Percent** | **Cumulative** | **Cumulative** |
|  |  |  | **Frequency** | **Percent** |  |  |  |  | **Frequency** | **Percent** |
| **Nulliparous or parous age ≥30 yr** | 225 | 25.92 | 225 | 25.92 |  | **Nulliparous or parous age ≥30 yr** | 1234 | 24.01 | 1234 | 24.01 |
| **Parous age <30 yr** | 643 | 74.08 | 868 | 100 |  | **Parous age <30 yr** | 3906 | 75.99 | 5140 | 100 |
| **Frequency Missing = 160** | | | | |  |  |  |  |  |  |

***Continued*. Distribution of key categorical variables before (left) and after (right) multiple imputations**

| **Menopausal status** | **Frequency** | **Percent** | **Cumulative** | **Cumulative** |  | **Menopausal status** | **Frequency** | **Percent** | **Cumulative** | **Cumulative** |
| --- | --- | --- | --- | --- | --- | --- | --- | --- | --- | --- |
|  |  |  | **Frequency** | **Percent** |  |  |  |  | **Frequency** | **Percent** |
| **Pre-/Peri-menopause** | 412 | 43.41 | 412 | 43.41 |  | **Pre-/Peri-menopause** | 2247 | 43.72 | 2247 | 43.72 |
| **Post-menopause** | 537 | 56.59 | 949 | 100 |  | **Post-menopause** | 2893 | 56.28 | 5140 | 100 |
| **Frequency Missing = 79** | | | | |  |  |  |  |  |  |
|  | | | |  |  |  |  |  |  |  |
| **Family history of breast cancer in 1st degree relatives** | **Frequency** | **Percent** | **Cumulative** | **Cumulative** |  | **Family history of breast cancer in 1st degree relatives** | **Frequency** | **Percent** | **Cumulative** | **Cumulative** |
|  |  |  | **Frequency** | **Percent** |  |  |  |  | **Frequency** | **Percent** |
| **No** | 791 | 82.22 | 791 | 82.22 |  | **No** | 4225 | 82.2 | 4225 | 82.2 |
| **Yes** | 171 | 17.78 | 962 | 100 |  | **Yes** | 915 | 17.8 | 5140 | 100 |
| **Frequency Missing = 66** | | | | |  |  |  |  |  |  |
|  | |  |  |  |  |  |  |  |  |  |
| **History of hysterectomy** | **Frequency** | **Percent** | **Cumulative** | **Cumulative** |  | **History of hysterectomy** | **Frequency** | **Percent** | **Cumulative** | **Cumulative** |
|  |  |  | **Frequency** | **Percent** |  |  |  |  | **Frequency** | **Percent** |
| **No** | 628 | 65.08 | 628 | 65.08 |  | **No** | 3389 | 65.93 | 3389 | 65.93 |
| **Yes** | 337 | 34.92 | 965 | 100 |  | **Yes** | 1751 | 34.07 | 5140 | 100 |
| **Frequency Missing = 63** | | | | |  |  |  |  |  |  |
|  | | |  |  |  |  |  |  |  |  |
| **History of bilateral oophorectomy** | **Frequency** | **Percent** | **Cumulative** | **Cumulative** |  | **History of bilateral oophorectomy** | **Frequency** | **Percent** | **Cumulative** | **Cumulative** |
|  |  |  | **Frequency** | **Percent** |  |  |  |  | **Frequency** | **Percent** |
| **No** | 845 | 86.84 | 845 | 86.84 |  | **No** | 4408 | 85.76 | 4408 | 85.76 |
| **Yes** | 128 | 13.16 | 973 | 100 |  | **Yes** | 732 | 14.24 | 5140 | 100 |
| **Frequency Missing = 95** | | | | |  |  |  |  |  |  |
